# Preclinical assessment of antigen-specific chimeric antigen receptor regulatory T cells for use in solid organ transplantation

**DOI:** 10.1038/s41434-022-00358-x

**Published:** 2022-08-05

**Authors:** Emma Proics, Marion David, Majid Mojibian, Madeline Speck, Nadia Lounnas-Mourey, Adeline Govehovitch, Wissam Baghdadi, Justine Desnouveaux, Hervé Bastian, Laura Freschi, Geoffrey Privat, Cédric Pouzet, Mauro Grossi, Pierre Heimendinger, Tobias Abel, David Fenard, Megan K. Levings, François Meyer, Céline Dumont

**Affiliations:** 1 Sangamo Therapeutics France, Valbonne, France; 2 BC Children’s Hospital Research Institute, Vancouver, BC Canada; 3 Department of Surgery, University of British Columbia, Vancouver, BC Canada; 4 School of Biomedical Engineering, University of British Columbia, Vancouver, BC Canada

**Keywords:** Kidney diseases, Transplant immunology, Cell delivery, Immunological techniques, Immunotherapy

## Abstract

A primary goal in transplantation medicine is the induction of a tolerogenic environment for prevention of transplant rejection without the need for long-term pharmacological immunosuppression. Generation of alloantigen-specific regulatory T cells (Tregs) by transduction with chimeric antigen receptors (CARs) is a promising strategy to achieve this goal. This publication reports the preclinical characterization of Tregs (TR101) transduced with a human leukocyte antigen (HLA)-A*02 CAR lentiviral vector (TX200) designated to induce immunosuppression of allograft-specific effector T cells in HLA-A*02-negative recipients of HLA-A*02-positive transplants. In vitro results demonstrated specificity, immunosuppressive function, and safety of TX200-TR101. In NOD *scid* gamma (NSG) mice, TX200-TR101 prevented graft-versus-host disease (GvHD) in a xenogeneic GvHD model and TX200-TR101 Tregs localized to human HLA-A*02-positive skin transplants in a transplant model. TX200-TR101 persisted over the entire duration of a 3-month study in humanized HLA-A*02 NSG mice and remained stable, without switching to a proinflammatory phenotype. Concomitant tacrolimus did not impair TX200-TR101 Treg survival or their ability to inhibit peripheral blood mononuclear cell (PBMC) engraftment. These data demonstrate that TX200-TR101 is specific, stable, efficacious, and safe in preclinical models, and provide the basis for a first-in-human study.

## Introduction

Solid organ transplantation requires lifelong immunosuppressive maintenance therapies, such as corticosteroids, calcineurin inhibitors (tacrolimus/cyclosporine), and mycophenolate mofetil to prevent allograft rejection. These treatments are associated with an increased risk of infections and de novo malignancies as complications of chronic immunosuppression, as well as drug-related adverse effects, reducing the life expectancy of transplant patients [1, 2]. Thus, the induction of a tolerogenic environment for prevention of immunological rejection of transplanted organs without the need for long-term pharmacological immunosuppression remains a primary goal in transplantation medicine.

Conditioning the immune response of solid organ transplant recipients towards allograft acceptance using cell-based therapies is clinically promising [3, 4]. Current efforts are focusing on regulatory T cells (Tregs), which are essential in controlling immune responses to alloantigens [3]. Tregs are involved in graft-specific tolerance after solid organ transplantation and exert their suppressive functions, such as inhibition of T- and B-cell proliferation and suppression of antigen-presenting cells, via a variety of cell contact-dependent and -independent mechanisms. Unlike pharmacological immunosuppressants, antigen-specific Treg therapy has the potential of generating specific immunotolerance targeted towards the allograft, without causing systemic immunosuppression [4].

Several clinical trials have been initiated to explore the use of Tregs in solid organ transplantation and other indications, including refined therapeutic approaches enabled by the development of flow cytometric, Good Manufacturing Practice-compliant protocols of Treg isolation [3, 4]. It has been well established that antigen-specific Tregs are more potent than polyclonal Tregs in preventing graft-rejection, offering targeted therapy rather than generalized immunomodulation [3]. One approach to generate allospecific Tregs is enrichment from a polyclonal population by ex vivo expansion upon allogeneic stimulation. Currently available preclinical and early-stage clinical trial results demonstrate the safety of polyclonal and antigen-specific Treg therapies in various settings [3]. The recently published ONE Study combined several cell-based therapies, including allospecific, donor-expanded Tregs, and compared them to standard of care in renal transplant patients [5]. Cell-based therapies were found to be safe and allowed reduction of immunosuppression to monotherapy in many cases, without affecting the allograft rejection rate significantly.

Genetic modification to generate specific, antigen-targeted Tregs is currently being explored in vitro and in vivo. Modification of T-cell receptor (*TCR*) alpha and beta chain genes to generate dual allospecificity has been found to protect allografts in a fully mismatched murine heart transplant model [3]. Another promising approach for generating antigen-specific Tregs is the use of chimeric antigen receptors (CARs) that combine antigen-binding domains, most commonly a single-chain variable fragment (ScFv) derived from the variable domains of antibodies, with the signaling domains of the TCR and additional costimulatory domains. This approach has already been successfully applied in oncology, to redirect conventional T cells (Tconvs) against tumors, leading to promising antitumoral activity in hematologic malignancies but also to severe and life-threatening adverse reactions [6]. Engineered antigen-specific Tregs were shown to be more potent than polyclonal Tregs in humanized mouse models of skin transplant [7–9] and GvHD [8, 10, 11] as well as in syngeneic mouse models [12, 13] clearly demonstrating the benefit of CAR-antigen specificity.

Mismatched human leukocyte antigens (HLAs) are an important barrier to successful transplantation. HLA molecules can elicit strong immune responses and generation of anti-donor HLA antibodies can lead to frequent rejection episodes [14, 15]. HLA class I molecule A*02 (HLA-A*02) has a high allelic frequency and a substantial proportion of organ transplantations will naturally result in a mismatch. Approximately 70% of Caucasian transplant recipients are HLA-A*02-negative and 30% of organ donors are HLA-A*02-positive, leading to a potential mismatch in 21% of transplantations [7, 16].

To generate immunotolerance, CARs can be employed to redirect naïve human Tregs towards a designated HLA class I molecule. We developed a humanized HLA-A*02 CAR for transduction of autologous Tregs from HLA-A*02-negative recipients of mismatched HLA-A*02-positive organs, with the aim to induce immunosuppression of allograft-specific effector T cells that might lead to graft rejection. Based on an initial HLA-A*02:01–specific CAR derived from the ScFv of a mouse monoclonal antibody against human HLA-A*02 [10], a panel of humanized HLA-A*02 CARs was developed and tested in Tregs for their ability to suppress xenogeneic graft-versus-host disease (GvHD) and rejection of human skin allografts in immunocompromised mice [7–9]. Based on these proof-of-concept experiments, we have further developed a lentiviral vector (TX200) encoding a CAR containing a humanized ScFv specific for the HLA-A*02 antigen, for use in a clinical setting, and optimized our gating strategy for isolation of autologous naïve (cluster of differentiation [CD] 45RA^+^) human Tregs (TR101). The resulting HLA-A*02 CAR-Tregs are named TX200-TR101.

A first-in-human trial is planned to investigate induction of allograft tolerance in HLA-A*02-negative recipients of a kidney transplant from an HLA-A*02-positive donor by administration of TX200-TR101 after transplantation. Notably, this will be the first time CAR-Tregs are investigated in a clinical trial. Therefore, we conducted a wide range of preclinical tests required for medicinal products containing genetically modified cells intended for use in humans, to assess the quality, safety, and efficacy of the cell product. This publication provides an overview of key components of the preclinical package.

## Materials and methods

### Ethics approval

All animal experiments were performed in accordance with relevant guidelines and regulations, either in an approved animal facility at Sangamo Therapeutics France (accredited by the French Ministry of Research, Arrêté n° 4566) according to the APAFIS (“*Autorisation de Projet utilisant des Animaux à des Fins Scientifiques*”)-approved protocol APAFIS#12909-2017121211476703 v4 and in compliance with the Guide for the Care and Use of Laboratory Animals, or at the University of British Columbia (UBC), approved by the UBC Animal Care Committee (A16-0300).

Experiments using human samples were performed in accordance with the Declaration of Helsinki and approved by appropriate ethics committees. Leukopaks for Treg isolation were sourced from HemaCare (Northridge, USA), an FDA-registered collection center, from healthy human volunteers who consented under an Institutional Review Board-approved protocol compliant with Code of Federal Regulations Title 21 Part 1271. Allogeneic T cells and peripheral blood mononuclear cells (PBMCs) were obtained from healthy donors following written consent according to protocols approved by the *Etablissement Français du sang*. Human skin discarded from plastic surgery was obtained from the Harvard Skin Resource Centre, Skin Works, or the Cambie Surgery Clinic according to protocols approved by the UBC Clinical Research Ethics Board (H16-02930).

### Generation and propagation of Tregs with HLA-A*02-specific CAR

The humanized HLA-A*02 ScFv was fused to a transmembrane domain and a signaling domain composed of the intracellular domain of human CD28 and CD3ζ. The control CAR TX235 was composed of the humanized HLA-A*02 ScFv and transmembrane domain but lacked intracellular signaling domains CD28 and CD3ζ. The resulting cDNA was cloned into a lentiviral vector. The TX200 lentivirus was produced by Lentigen/Miltenyi (Gaithersburg, USA). The TX235 lentivirus was generated in house by transfecting adherent HEK293T cells (Lenti-X) with a lentiviral 4-plasmid system including human immunodeficiency virus type 1 (HIV-1) gagpol, HIV-1 Rev, envVSV-G, and the TX235 CAR transfer plasmid. A schematic of TX200 and TX235 is presented in the supplementary data (Fig. S1). Surface expression was determined by flow cytometry using fluorescent HLA-A*02 dextramer (#WB2666, Immudex, Denmark).

White blood cells were harvested from healthy HLA-A*02-negative donors using leukapheresis and were cryopreserved (HemaCare). The leukapheresate was thawed and cells were isolated after staining for CD4 (PE-Vio770, #130-093-142), CD45RA (FITC, #170-076-502), CD25 (PE, #170-076-505) and CD127 (APC, #170-076-501) with antibodies that were of Good Manufacturing Practice-grade, except CD4 PE-Vio770, (all Miltenyi Biotech) to isolate naïve Tregs defined as CD4^+^/CD45RA^+^/CD25^high^/CD127^low^ using an SH800 cell sorter (Sony, Japan). The gating strategy is shown in the supplementary data (Fig. S2). Cells were then resuspended in X-VIVO-15 medium (#BEBP02-054Q, Lonza) with 1000 U/ml interleukin (IL)-2 (#2238131-A, Novartis Pharma) and activated with anti-CD3/CD28-coated dynabeads (#40203D, Life Technologies). After 72 h, cells were transduced with the lentiviral vector and further expanded in X-Vivo-15 medium with 1000 U/ml IL-2 and anti-CD3/CD28-coated dynabeads. Medium was refreshed with IL-2 every 2 days. Cells were expanded for less than 2 weeks to keep the cells in an exponential phase of growth and minimize loss of the naïve Treg phenotype. At harvesting, magnetic beads were removed with the CTS™ DynaMag system (#12102, ThermoFisher, France) and cells were washed and cryopreserved in AT2 vials (AT-Closed Vial^®^, Aseptic Technologies, Belgium) in CryoStor CS10 (#210102, BioLife Solutions, USA). Vials were stored at −150 °C in a dry freezer until further use. After lentiviral transduction and expansion, cells were 100% CD4^+^/CD45RA^+^, 99.8 ± 0.10% CD25^+^, 97.8 ± 0.59% CD127^low^, and 93.6 ± 1.26% FOXP3^+^ (mean ± SEM with *n* = 16), with FOXP3 TSDR hypomethylation of 89.1 ± 3.4% after thawing. Cells were viable at 86.9 ± 1.3% and transduced with the HLA-A*02 CAR at 54.7 ± 1.8% (mean ± SEM, *n* = 20). Of note, an integration site analysis indicated polyclonal insertion of the lentiviral vector and no unexpected integration or site selectivity were found (data not shown).

### Flow cytometry

Cells from in vitro experiments were washed with phosphate-buffered saline (PBS)/4% bovine serum albumin and stained for cell surface markers including HLA-A*02 dextramer (#WB2666-APC, Immudex, Denmark), anti-human CD4, and anti-human CD69 (VioBlue, #130-113-219 and APC-Vio770, #130-112-616, Miltenyi Biotech, France). Prior to in vivo experiments, the number of HLA-A*02 CAR-expressing cells in the Treg batch used was determined using HLA-A*02 dextramer (#WB2666-PE, Immudex, Denmark).

Following in vivo experiments, spleen and brain samples were passed through a 70-µm cell strainer to obtain a single cell suspension. Brain cells were then incubated in RPMI/5% fetal calf serum (FCS) with 2.5 mg/ml collagenase D (#11088858001, Sigma–Aldrich, France) at 37 °C under agitation, washed in RPMI/5% FCS, and centrifuged. The cell pellet was resuspended in 70% (v/v) Percoll solution (#GE17-0891-01, Sigma–Aldrich, France), centrifuged at 500 × *g* over 37% (v/v) Percoll, and cells at the interface of the Percoll 70/37 gradient were recovered. Other tissues were roughly chopped with a scalpel blade and digested in RPMI/5% FCS containing 150 µg/ml collagenase D and 25 µg/ml DNase I (#10104159001, Sigma–Aldrich, France) and passed through a cell strainer. Red blood cells in blood samples and tissues were lysed with Red Blood Cell Lysing Buffer (#R7757, Sigma–Aldrich, France) or ammonium chloride. Blood and tissue cells were washed with PBS/2% FCS and incubated with mouse Fc block (#553142, BD Biosciences, France). Cells were then washed with PBS/4% bovine serum albumin and stained. Fixable viability dyes eFluor780 or eFluor506 (#65-0865-14 and #65-0866-14, ThermoFisher, France) were used. Cells were stained first with anti-human HLA-A*02 (BV421, #740082 or PE, #558570, BD Biosciences, France) and other cell surface markers, including anti-human CD4 (PE-Cy7, #557852 or FITC, #555346, BD Biosciences, France; or Percp-Cy5.5, #45-0049-42, ThermoFisher, France) and anti-human CD45 (VioGreen, #130-110-638, Miltenyi Biotech, France), and then fixed and permeabilized with the forkhead box P3 (FOXP3) staining buffer set (#00-5523-00, ThermoFisher, France), and stained for intracellular markers, including anti-human FOXP3 (PE, #12-4777-42, ThermoFisher, France or Alexa Fluor 647, #560045, BD Biosciences, France). Further antibody details are provided in the supplementary methods.

An Attune™ NxT flow cytometer and Attune™ NxT software were used for in vitro experiments, the biodistribution study, and tacrolimus study and analyses were performed with FlowJo V10. A MACSQuant Analyzer 10 with MACSQuantify software 2.8 was used for the GvHD model and hypomethylation study, and Cytoflex (Beckman Coulter) with FlowJo Software V9.9.4 and 10.3 was used for the skin transplant experiment. Naïve Tregs were isolated using an SH800 cell sorter and the gating strategy was modified from [17]. Exemplary gating strategies are presented in the supplementary methods (Fig. S2).

### *FOXP3* hypomethylation

To assess *FOXP3* Treg-specific demethylated region (TSDR) hypomethylation, genomic DNA was extracted from cell pellets using the DNeasy Blood and tissue kit (#69506, Qiagen, USA), DNA bisulfite conversion was done using the Epitech Fast Bisulfite Conversion Kit (#59826, Qiagen, USA), and *FOXP3* TSDR hypomethylation was then assessed by qPCR high-resolution melting curve analysis using Precision Melt Supermix (#1725112, Biorad, France) and *FOXP3* TSDR-specific primers (forward: TTGGGTTAAGTTTGTTGTAGGATAG, reverse: ATCTAAACCCTATTATCACAACCCC, Sigma Aldrich, France). The fragment analyzed included 11 CpG sites and total TSDR methylation was calculated as the mean of methylation percentage of each individual CpG. Methylation levels were determined using a standard curve (#59695, Qiagen). For CAR-Treg products manufactured from leukapheresates of female donors, raw values were corrected to consider that one of the two TSDR alleles is fully methylated as a result of X-inactivation.

### Plasma cytokine assessment

Human cytokines in plasma (interferon-γ [IFNγ], IL-1β, IL-2, IL-6, IL-10, IL-12p70, IL-13, IL-17A/F) were measured using multiplex cytokine immunoassay plates (V-plex custom human biomarker proinflammatory panel, #30098491, MesoScaleDiscovery, USA) according to manufacturer’s instructions.

### In vitro assays

For in vitro experiments, each Treg donor constituted an individual experiment and n represents the number of Treg donors. Per experiment, 3 to 4 different Treg batches were used (see figure legends for number of biological and technical replicates). Mycoplasma testing was performed for all isolated cells as part of the quality assessments.

For activation, suppression, and cross-reactivity assays, TX200-TR101 Tregs were first cultured at 37 °C in X-VIVO-15 medium (#BEBP02-054Q, Lonza) with 300 IU/ml IL-2 (#2238131-A, Novartis Pharma) for 48 h.

In the activation assay, TX200-TR101 Tregs were stimulated for 24 h with anti-CD3/CD28-coated dynabeads (#40203D, Life Technologies, 1 bead:1 cell ratio) as a positive control for maximum TCR activation, with HLA-A*02-positive PBMCs (1:1 ratio of PBMCs to Tregs), with HLA-A*02 dextramer (#WB2666-PE, Immudex, Denmark), or with HLA-A*02-negative PBMCs (1:1 ratio of PBMCs to Tregs) or blank dextramer (#NI3233, Immudex, Denmark) as negative controls. Cells were then harvested and analyzed by flow cytometry.

In the suppression assay, Tconv were stained with a fluorescent proliferation dye (eFluor450, #65-0842-85, ThermoFisher, France). TX200-TR101 Tregs were cultured without IL-2 for 24 h and preactivated with HLA-A*02 or blank dextramer for 24 h while allogeneic CD4^+^/CD25^–^ Tconv were preactivated for 24 h with anti-CD3/CD28-coated dynabeads (1:1 ratio). Preactivated Tconv and allogeneic TX200-TR101 Tregs were then co-cultured at Tconv:Treg ratios of 1:1, 2:1, 4:1, 8:1, 16:1 or 1:0 for 3 days at 37 °C in X-VIVO-15 medium. Proliferation suppression of Tconv was assessed by flow cytometry. The percentage of suppression was calculated for each ratio Tconv:Treg as the percentage of inhibition of the Tconv proliferation when cultured with Tregs compared with Tconv cultured alone.

In the cross-reactivity assay, TX200-TR101 Tregs were co-cultured with characterized cryopreserved human PBMCs (#CTL-UP1, Cellular Technology Limited) in a 1:1 ratio for 24 h. The HLA genotypes of the PBMCs that were available in the biobank for testing are specified in the supplementary. Cells were then harvested and analyzed by flow cytometry.

To assess the risk of cellular transformation and acquisition of a tumorigenic potential, TX200-TR101 Tregs cultured in X-VIVO-15 medium with 300 IU/ml IL-2 for 24 h were left unstimulated or chronically stimulated with anti-CD3/CD28-coated dynabeads (1:1 ratio) in the presence or absence of high-dose IL-2 (1000 IU/ml) for up to 75 days. Expansion with chronical stimulation was performed every 7 days and sub-passage every 2 days until cell death. From the third round of activation with beads, and/or depending on the cell growth, beads were replaced every 7 days. Three days after activation, cells were counted and diluted with fresh medium. This medium refresh was performed every 2 days, based on cell counting. In culture conditions where viability reached <3–5%, cells were kept in culture for an additional 15 days, with a medium refresh every 2–3 days, to ensure no clone of immortalized cells would grow. For cells cultured with high-dose IL-2, several starvation cycles were performed to ensure that no IL-2-independent cell growth occurred. Weekly, 2 samples of 1–0.5 × 10^6^ cells were frozen in 10% dimethyl sulfoxide and sent to Life Length^®^ (Madrid, Spain) for assessment of telomere length using high throughput quantitative fluorescent in situ hybridization technology [18] and of telomerase activity using a telomerase repeat activity protocol modified for real-time qPCR analysis [19]. Each assay analyzed 5 technical replicates.

### Mouse models

NOD *scid* gamma (NSG) mice (NOD.Cg-*Prkdc*^*scid*^ *Il2rg*^*tmWjl*^ /SzJ, JAX stock no 005557) [20] and HLA-A*02 NSG mice with humanized HLA-A*02 (NOD.Cg-*Prkdc*^*scid*^ *Il2rg*^*tm1Wjl*^ Tg[HLA-A/H2-D/B2M]1Dvs/SzJ, JAX stock no 014570) [21] were purchased from Jackson Laboratory (Bar Harbor, USA), distributed by Charles River (Lyon, France) or bred in house at the UBC. Unless otherwise specified, mice were housed in a specific opportunistic pathogen-free facility, in individually, positively ventilated polysulfone cages with HEPA-filtered air, controlled 12 h light/dark cycle, temperature of 20–26 °C, and relative humidity of 30–70%. Filtered tap water and standard rodent chow were provided ad libitum. For experiments, mice were age-matched but distributed randomly to treatment groups. Except for the skin graft experiment that was conducted with two Treg batches, each in vivo experiment was conducted with one Treg batch/donor and n represents the number of mice per group.

Unless otherwise specified, mice were conditioned with intraperitoneal injection of 30 mg/kg busulfan (#B2635, Sigma–Aldrich) to favor human cell engraftment approximately 24 h prior to intravenous human cell injection via the tail vein. The number of cells for injection was determined for each batch of Tregs by flow cytometric analysis of HLA-A*02 CAR-expressing cells using dextramer. The percentage of dextramer-positive cells ranged from 45–72% and the number of Tregs injected ranged from 2.5–5 × 10^6^ cells per mouse in the individual experiments. *FOXP3* hypomethylation levels were also assessed prior to cell injection. Throughout experiments, body weights and GvHD scores were monitored 3 times weekly by investigators that were blinded to treatment. GvHD was scored based on weight, fur texture, posture, activity level, and skin integrity. Unless otherwise specified, categories were rated from 0–3 as described in the supplementary (Table S2), with higher scores indicating more symptoms. During experiments, blood samples were collected by retro-orbital sampling under local anesthesia (unless specified otherwise below). At the end of experiments or when ethical endpoints were reached, mice were euthanized by cervical dislocation, necropsy was performed, and tissue and blood samples were collected.

In the GvHD experiment, a total of 30 female NSG mice aged 7–8 weeks and with a median (range) weight of 22 g (20–25 g) were randomly assigned to one of the 3 following groups: HLA-A*02-positive human PBMCs alone, together with TX200-TR101 Tregs, or with TX235-TR101 Tregs (*n* = 5 per group/2 pooled experiments). Mice were conditioned with busulfan, injected with HLA-A*02-positive human PBMCs and/or Tregs (1:1 ratio, 5 × 10^6^ cells each), and monitored as described above for 4 weeks. Blood samples for flow cytometry were collected weekly and blood and spleen were collected at sacrifice. Representative fluorescence-activated cell sorting (FACS) plots obtained in blood are shown in the supplementary data (Fig. S3).

In the hypomethylation experiment, a total of 11 male and female HLA-A*02 NSG mice aged 8–11 weeks and with a median (range) weight of 29 g (23–36 g) were randomly assigned to one of the 3 following groups according to the degree of *FOXP3* TSDR hypomethylation of the injected TX200-TR101 Tregs: 80.8% hypomethylation (*n* = 5, 3 males and 2 females), 92% (*n* = 3, 1 male and 2 females), and 89.1% (*n* = 3, 2 males and 1 female). Mice were conditioned with busulfan, injected with TX200-TR101 Tregs (2.5 × 10^6^ cells per mouse), and monitored as described above for 27 days. Blood samples for flow cytometry were collected weekly and blood, spleen, liver, and lung were collected at sacrifice. The results of these experiments are presented together with results of previous experiments with a similar set-up, where Tregs isolated with a previous gating strategy (TR100) were included. In these experiments, male and female HLA-A*02 NSG mice aged 8–11 weeks received TX200-TR100 Tregs with 69% hypomethylation (*n* = 20, 9 males and 11 females), or TX200-TR101 Tregs with 100% hypomethylation (*n* = 5, 2 males and 3 females) or 98% hypomethylation (*n* = 2, 1 male and 1 female), and were monitored for up to 27 days.

In the biodistribution experiment, a total of 24 male and female HLA-A*02 NSG mice aged 8-10 weeks and with a median (range) weight of 24 g (20–31 g) were conditioned with busulfan, injected with TX200-TR101 Tregs (3 × 10^6^ cells per mouse), and monitored as described above. Mice were randomly assigned to one of 3 sacrifice timepoints at 1, 2, and 3 months (*n* = 8, 3 males and 5 females, planned per timepoint). Blood samples for flow cytometry were collected weekly and blood (for flow cytometry and plasma cytokine assessment), spleen, liver, lung, kidney, heart, testis/ovaries, brain, and intestine were collected at sacrifice. Gross pathology and histopathology were performed. For animals euthanized 2 and 3 months post-injection, parts of the spleen, lung, kidney, liver, testis/ovary, brain, and the whole intestine were collected for histopathology. Any gross lesion/abnormal tissue growth during the study was collected with half the lesion analyzed by flow cytometry and half fixed in 10% buffered formalin for histological analysis. The rest of the spleen, liver, lung, kidney, heart, brain, and testis/ovaries were processed for flow cytometric analysis. The gating strategy is shown in the supplemental data (Fig. S4).

In the tacrolimus experiment, a total of 20 female NSG mice aged 7 weeks and with a median (range) weight of 21 g (17–25 g) were randomly assigned to one of the 4 following treatment groups: PBMCs only (*n* = 7), PBMCs plus tacrolimus (*n* = 6), TX200-TR101 Tregs (*n* = 3), and TX200-TR101 Tregs plus tacrolimus (*n* = 4). Mice were conditioned with busulfan and injected with PBMCs and/or Tregs (5 × 10^6^ cells each), as described above and tacrolimus groups received daily intraperitoneal injections of 0.5 mg/kg tacrolimus (Prograf, #29485063, Astellas Pharma, through Euromedex, France) for 3 weeks, starting 7 days after cell injection. The tacrolimus dose was chosen based on the results of pilot studies, where doses between 0.1 and 2 mg/kg had been tested. Mice were monitored as described above for a total duration of 3 weeks. Spleen was collected at sacrifice.

In the skin transplant experiment, a total of 32 female NSG mice aged 8–12 weeks and with a median (range) weight of 22 g (20–25 g) received dorsal skin transplants of approximately 1 cm^2^ from human HLA-A*02-positive donors as described previously [8]. Nineteen mice received a graft from Donor 1 (Cohort 1) and 13 mice received a graft from Donor 2 (Cohort 2). Nine weeks after skin transplantation, mice were randomly assigned to one of the 4 following groups: PBS (*n* = 3 each for Cohorts 1 and 2), HLA-A*02-negative human PBMCs (*n* = 6 for Cohort 1, *n* = 3 for Cohort 2), HLA-A*02-negative human PBMCs plus autologous TX200-TR101 Tregs (*n* = 7 for Cohort 1, *n* = 5 for Cohort 2), and TX200-TR101 Tregs alone (*n* = 3 for Cohort 1, *n* = 2 for Cohort 2) and injected with 10 × 10^6^ PBMCs and/or 5 × 10^6^ Tregs (2:1 ratio). Each cohort received a different batch of TX200-TR101 (Donors 1 and 2). Human PBMC engraftment and skin rejection were analyzed and blood samples for flow cytometry collected weekly. GvHD score categories in this experiment were rated from 0–2 as described in Cooke et al. 1996 [22] and blood samples were collected from the saphenous vein. The mice were monitored for 28 to 35 days, and skin graft, surrounding mouse skin, spleen, intestine, liver, and lung were harvested for histopathology at sacrifice. GvHD and skin rejection scores of both cohorts were pooled for analysis.

### Histology and immunohistochemistry

Human skin grafts and surrounding mouse skin were fixed overnight at 4 °C in 10% formalin and stored in 70% ethanol before paraffin-embedding and preparation of hematoxylin/eosin-stained sections. For histopathological skin graft scoring, slides were evaluated by a blinded clinical pathologist using a scoring system defined by 6 factors (Lerner grade, spongiosis, necrotic keratinocytes, adnexal involvement, parakeratosis, lymphoid cuffs in dermis) as described previously [8]. Tissues collected in the biodistribution study were fixed in 10% buffered formalin and shipped to Vetopath (Antibes, France) for histological analysis. Tissues were dehydrated in Tissue Tek VIP2000 (Miles Scientific) and embedded in resin-mixed paraffin after xylene baths. Tissues collected at the UBC were shipped to Pacific Tox Path, LLC (USA) for evaluation, where tissues were trimmed and processed to paraffin blocks. All tissue blocks were cut at 4–5 µm and sections stained with hematoxylin/eosin and examined microscopically by a board-certified veterinary pathologist.

Immunohistochemistry for detection of human FOXP3 and human CD45 in mouse organs (intestine, liver, lung, and spleen) and human skin grafts and analysis of slides was performed as described previously [8]. Immunohistochemical localization of CD45 as leukocyte marker and FOXP3 as Treg marker was performed by light microscopy for individual mice and scored according to a grading scale: 0 (no findings), 1 (minimal), 2 (mild), 3 (moderate), 4 (marked) and 5 (severe).

### Statistics

Sample size calculation for mouse experiments was performed with GPower software 3.1. Five mice per group were needed to get an effect size of 2 between control PBMC- and CAR-Treg-treated groups with a test power of 0.89. Statistical analyses were conducted using GraphPad Prism 8.4.3. Information on sample size, replicates, variables, and statistical significance testing is provided in the figure legends. One-way ordinary analysis of variance (ANOVA), 2-way ANOVA (with Tukey’s or Dunnett’s multiple comparison test), Kruskal–Wallis multiple comparison test with Dunn’s post-test, or Holm-Sidak method following multiple t-test, were performed as appropriate to assess statistical significance. *P*-values < 0.05 were considered statistically significant. *P*-values in figures are presented as *p < 0.05, **p < 0.01, ***p < 0.001, and ****p < 0.0001.

## Results

### TX200-TR101 Tregs are HLA-A*02-specific and exert immunosuppressive function in vitro

The clinical candidate TX200 was developed based on the humanized HLA-A*02 ScFv variant identified in the proof-of-concept study [8], with a CAR harboring an ScFv from a humanized antibody against HLA-A*02 linked to protein-subunits composed of CD28 and CD3ζ. An enriched subset of naïve specific CD4^+^/CD45RA^+^/CD25^high^/CD127^low^ Tregs (TR101) transduced with the HLA-A*02-specific CAR construct (TX200-TR101 Tregs) was first characterized in vitro.

After lentiviral transduction and expansion, cells were 100 ± 0% CD4^+^/CD45RA^+^, 99.8 ± 0.10% CD25^+^, 97.8 ± 0.59% CD127^low^, and 93.6 ± 1.26% FOXP3^+^ (mean ± SEM, *n* = 16). Quantification of cytokines in the culture supernatants measured by multiplex cytokine immunoassay showed that the Tregs produced only IFNγ (mean ± SEM of 967.4 ± 442.1 pg/ml after TCR stimulation [*n* = 12] and 171.1 ± 40.1 pg/ml after CAR stimulation [*n* = 12]) in vitro. Levels of IL-2, IL-17A and IL-4 were below the lower limit of quantification after activation either through their TCR by anti-CD3/CD28-coated dynabeads or through their CAR using HLA-A*02 dextramer. These data indicated that transduction and expansion of TX200-TR101 did not alter the Treg phenotype.

**Fig. 1:**
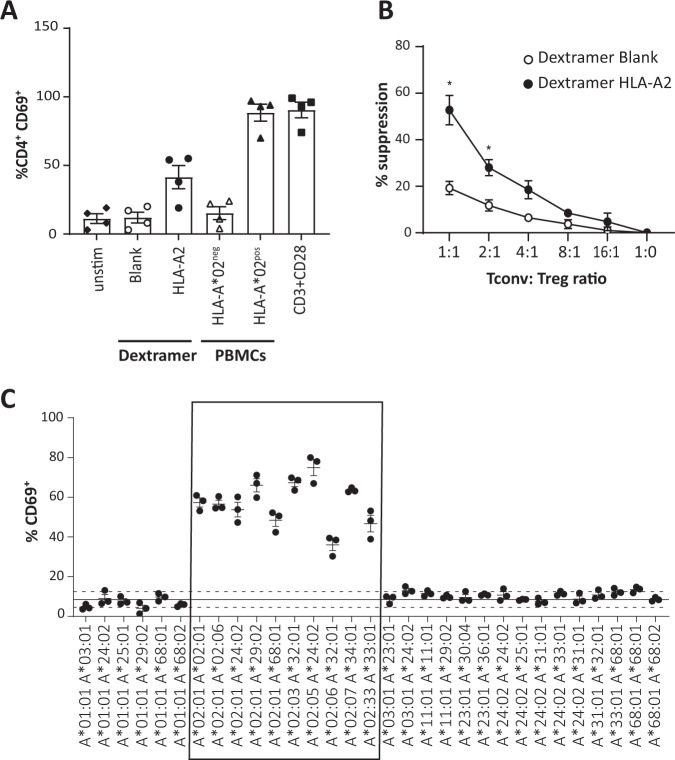
In vitro characterization of naïve Tregs transduced with the HLA-A*02 CAR (TX200-TR101 Tregs). **A** TX200-TR101 Tregs were activated using either HLA-A*02 dextramer, blank dextramer (negative control), HLA-A*02-positive human PBMCs, HLA-A*02-negative PBMCs (negative control), or anti-CD3/CD28-coated dynabeads as a positive control with maximum-level polyclonal TCR activation. Treg activation was monitored via CD69 activation in CD4^+^ cells by flow cytometry. Bars represent mean ± SD (*n* = 4 Treg batches). The percentage of transduction of HLA-A*02 CAR was 59.2 ± 3.1% (mean ± SEM). **B** TX200-TR101 Tregs pre-activated with HLA-A*02 dextramer or control dextramer were co-cultured with allogeneic CD4^+^/CD25^–^ T cells (Tconv), preactivated with anti-CD3/CD28-coated dynabeads for 3 days, at Tconv:Treg ratios of 1:1, 2:1, 4:1, 8:1, 16:1, or 1:0. Tconv suppression was assessed by flow cytometric analysis following staining with the fluorescent proliferation dye eFluor450. Data are presented as mean ± SEM (*n* = 4 Treg batches). The percentage of transduction of HLA-A*02 CAR was 49 ± 2.9% (mean ± SEM). Statistical significance of data was determined using the Holm-Sidak multiple t-test method (*p < 0.05). **C** Specificity of TX200-TR101 Tregs was assessed by co-culture with human PBMCs with various HLA-A alleles (genotypes shown on the *X*-axis). Treg activation was monitored via the percentage of CD69^+^ cells by flow cytometry. Data are presented as scatter plots, with each dot representing a Treg batch and lines representing the mean ± SEM (*n* = 3 Treg batches, 1 technical replicate). The percentage of transduction of HLA-A*02 CAR was 56 ± 2.3% (mean ± SEM). The continuous line represents the baseline of activation of non-transduced Tregs and the dotted lines the variation ± 2 SD from the mean. The box highlights PBMCs positive for HLA-A*02 allele(s).

Specificity of activation of TX200-TR101 Tregs through the CAR by the target antigen HLA-A*02 was demonstrated by flow cytometric monitoring of CD69, one of the earliest cell surface antigens upregulated following Treg activation [23], in CD4^+^ cells (Fig. 1A). Stimulation of TX200-TR101 Tregs with HLA-A*02-positive PBMCs resulted in an activation level comparable to anti-CD3/CD28-coated dynabeads that were used as a positive control for maximum activation via the TCR (88.5 ± 6.2% CD4^+^/CD69^+^ cells for PBMC-stimulated cells versus 90.5 ± 5.6% CD4^+^/CD69^+^ for TCR-stimulated cells [mean ± SEM, *n* = 4]); activation was also observed with HLA-A*02 dextramer (41.5 ± 8.4% CD4^+^/CD69^+^ cells), whilst blank dextramer and HLA-A*02-negative PBMCs did not lead to an increase in CD4^+^/CD69^+^ cells (12.0 ± 3.9% and 15.2 ± 4.7% CD4^+^/CD69^+^ cells, respectively).

The ability of TX200-TR101 Tregs to suppress allogeneic Tconv was assessed after co-culture at different ratios following pre-activation of TX200-TR101 Tregs with HLA-A*02 dextramer and of Tconv with anti-CD3/CD28-coated dynabeads (Fig. 1B). TX200-TR101 Tregs preactivated by their target antigen HLA-A*02 (HLA-A*02 dextramer) exerted a dose-dependent suppressive effect on Tconv proliferation, with 52.7 ± 6.3% suppression observed when co-cultured in a 1:1 ratio versus 19.2 ± 2.8% suppression for Tregs activated with control dextramer blank (mean ± SEM, *n* = 4).

Due to a high degree of homology between HLA alleles, virtually all anti-HLA antibodies have some cross-reactivity. To ensure safety and bioavailability of the clinical candidate, cross-reactivity towards other HLA-type molecules should be minimal whilst ideally every sub-allele of HLA-A*02 should activate the TX200-TR101 Tregs. Specificity was assessed by co-culture of TX200-TR101 Tregs with characterized, cryopreserved human PBMCs followed by flow cytometric assessment of CD69^+^ cells. The *HLA-A* alleles of all assessed PBMCs as well as their genotype regarding other HLA genes, such as *HLA-B*, *HLA-C*, *HLA-DRB1*, *HLA-DQB1*, *HLA-DPB1*, *HLA-DQA1*, and *HLA-DPA1*, are provided in the supplementary (Table S3). TX200-TR101 Tregs were activated by co-culture with all PBMCs carrying HLA-A*02-alleles (36–75% CD69^+^ cells), and 1 allele of HLA-A*02 was sufficient to induce activation, whilst only very few Tregs were activated by co-culture with PBMCs carrying other *HLA* alleles (4–13% CD69^+^) (Fig. 1C). Of note, no cross-reactivity was observed against HLA-A*68 and HLA-A*25, two alleles that were recognized by the non-humanized form of this CAR [8]. Testing against HLA-A*69, an allele highly similar to HLA-A*02 which would be expected to activate this CAR, was not done as this genotype is very rare and HLA-A*69-positive PBMCs could not be sourced. In the upcoming clinical trial, recipients that are HLA-A*69-positive will be excluded to ensure patient safety. Similar results were observed with other activation markers (see Supplementary Table S3).

### TX200-TR101 Tregs are efficacious in a mouse model of xenogeneic GvHD

**Fig. 2:**
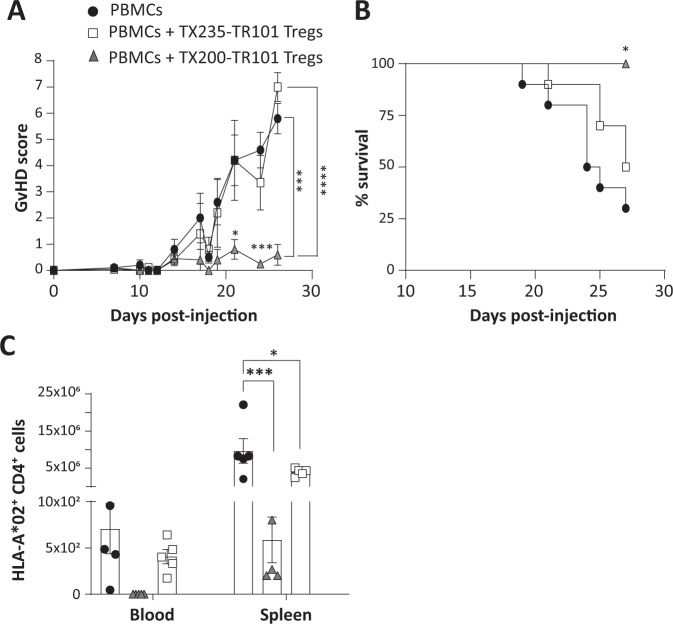
HLA-A*02-specific CAR-Tregs are efficacious at preventing GvHD onset. 7- to 8-week old female NSG mice were injected intravenously with HLA-A*02-positive human PBMCs with or without HLA-A*02-specific CAR-Tregs, that were either transduced with the TX200 construct (TX200-TR101 Tregs; percentage of transduction: 50.2%) or with a control CAR with non-signaling endodomain (TX235-TR101 Tregs; percentage of transduction: 71%). Mice were injected with 5 × 10^6^ CAR-Tregs (**A**) GvHD score over time, with data presented as mean ± SEM (*n* = 5 per group, scoring in 5 categories with 0: no findings, 1: mild, 2: moderate, 3: severe combined into a total score). Statistical significance was determined using 2-way ANOVA: *p < 0.05. **B** Survival curve, Log-rank (Mantel-cox) test with *p = 0.039. **C** PBMC engraftment in blood and spleen, 4 weeks after injection according to flow cytometric assessment of CD4^+^ HLA-A*02-positive cells. Data are shown as dot plots, with each dot representing a mouse and bars representing the mean and error bars the SEM. For blood, the number of CD4^+^ cells/µl blood is shown. Statistical significance was determined using 2-way ANOVA with Tukey’s multiple comparison test: *p < 0.05, ***p < 0.001.

Two mouse models were used to test the capacity of TX200-TR101 Tregs to reduce effector T cell activity in vivo. NSG mice were used as a xenogeneic GvHD model. These mice have been shown to accept the engraftment of mature human PBMCs for a limited time, until GvHD onset around 3 to 4 weeks post-administration due to cytotoxic and proinflammatory effects of activated CD4^+^ and CD8^+^ Tconvs [24]. NSG mice conditioned with busulfan were injected with HLA-A*02-positive human PBMCs alone or in combination with TX200-TR101 Tregs, or with negative control Tregs transduced with a control CAR (TX235-TR101). These TX235-TR101 Tregs are equivalent to polyclonal Tregs as this control CAR is devoid of a signaling domain. Mice injected only with PBMCs or co-injected with TX235-TR101 Tregs displayed significant GvHD, quantified as increased GvHD scores (mean ± SEM 5.8 ± 0.58 and 7.0 ± 0.55, respectively, at day 26), whilst scores remained low in mice co-injected with TX200-TR101 Tregs (mean ± SEM 0.6 ± 0.4 at day 26) (Fig. 2A). Survival of mice receiving TX200-TR101 Tregs was significantly improved compared to mice receiving PBMCs only or control Tregs TX235-TR101 (Mantel-Cox Log-rank *p* = 0.039) although the onset of GvHD was slightly delayed in this control group (median survival 24.5 days for mice injected with PBMCs versus 27 days for mice injected with PBMCs plus TX235-TR101 control Tregs) (Fig. 2B). Human PBMC engraftment and proliferation in blood and spleen, as assessed by the presence of human CD4^+^ HLA-A*02-positive cells, was reduced in mice co-injected with TX200-TR101 Tregs (mean ± SEM 0.57 ± 0.22 CD4^+^ HLA-A*02-positive cells/µl of blood versus 706.1 ± 268.8 for mice injected with PBMCs only and 406.1 ± 77.3 for mice injected with PBMCs plus TX235-TR101 control Tregs), with a statistically significant difference observed in spleen (9.62 × 10^6^ ± 3.33 × 10^6^ CD4^+^ HLA-A*02-positive cells per spleen for mice injected with PBMCs only versus 586.67 ± 247 in mice injected with PBMCs plus TX200-TR101) (Fig. 2C). Co-injection of Tregs carrying the control CAR TX235 resulted in a slight reduction of engraftment (3.92 × 10^6^ ± 4.54 × 10^5^ CD4^+^ HLA-A*02-positive cells in spleen).

### TX200-TR101 Tregs localize to skin grafts in a human skin transplant mouse model

The functional capacity of the TX200-TR101 batches used in the experiment was tested in a GvHD model, where they were shown to effectively prevent GvHD onset in NSG mice (see Supplementary Fig. S5).

**Fig. 3:**
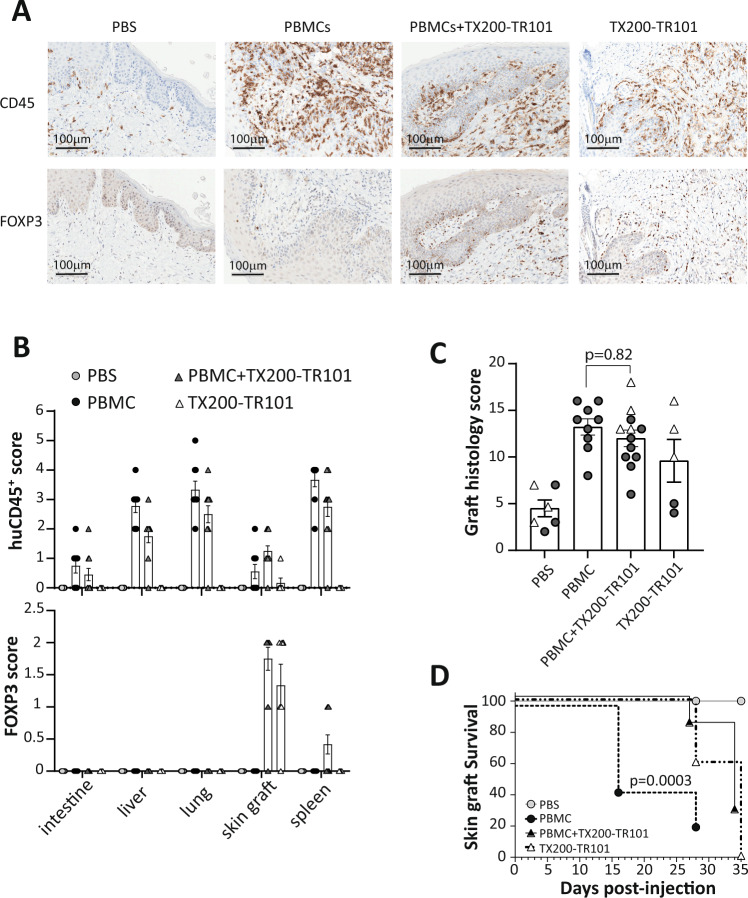
HLA-A*02-specific CAR-Tregs relocalize in skin grafts but have a minimal effect on human skin graft rejection. 8- to 12-week old female NSG mice were transplanted with skin from human HLA-A*02-positive donors and co-injected with human HLA-A*02-negative PBMCs and/or autologous TX200-TR101 Tregs at a PBMC:Treg ratio of 2:1. Controls were injected with PBS. Two batches of Tregs were used with 52 and 48% of HLA-A*02 CAR transduction, respectively. **A** Transplanted skin grafts harvested at day 28 were immuno-stained for CD45 and FOXP3 to show the proportion of human CD45^+^ and FOXP3^+^ cells in each section. Representative images are shown; scale bars: 100 µm. **B** Upper and lower panel show immunohistochemical assessment of human CD45 and FOXP3, respectively, in different mouse tissues and human skin grafts 4 weeks after injection. Bars represent means ± SEM (*n* as above, immunohistochemistry scoring 0: no findings, 1: minimal, 2: mild, 3: moderate, 4: marked, 5: severe); absence of bars indicates cells were not detectable. **C** Cumulative skin graft histology scores 28 and 35 days after injection, presented as pooled results from 2 individual experiments. In each experiment (cohort), mice were engrafted with one batch of skin and injected with one batch of cells. Data are presented as a scatter-plots with grey circles showing day 28 scores and white triangles showing scores at day 35, with each dot representing a mouse, bars representing the mean of the pooled day 28 and day 35 data, and error bars the SEM of the pooled data (*n* = 6 for PBS, *n* = 9 for PBMCs, *n* = 12 for PBMC plus TX200-TR101, *n* = 5 for TX200-TR101). Statistical significance of data was calculated using 1-way ANOVA with multiple comparison to the PBMC group. **D** Skin graft survival curve, log-rank (Mantel-Cox) test. For the PBMC group, all mice were terminated at day 28 due to onset of GvHD.

To assess whether TX200-TR101 Tregs could prevent allograft rejection in vivo, NSG mice received skin transplants from human HLA-A*02-positive donors, followed by injection of HLA-A*02-negative human PBMCs to elicit skin graft rejection, with or without autologous TX200-TR101 Tregs, or TX200-TR101 Tregs alone or PBS as controls. Immunostaining of CD45 to detect human T cells and of FOXP3 to detect human Tregs in skin grafts showed human cells infiltrating in skin grafts of mice injected with PBMCs, PBMCs plus TX200-TR101, and TX200-TR101 Tregs, whilst FOXP3^+^ cells were detected only in skin of mice injected with PBMCs plus TX200-TR101 and TX200-TR101 Tregs alone (Fig. 3A).

Human T cells, indicated by qualitative assessment of human CD45 immunopositivity, were detected at low levels in the intestine (mean ± SEM score 0.75 ± 0.25 arbitrary units [AU] as estimated according to the grading score scale shown in the methods section), and at higher levels in liver (2.78 ± 0.22 AU), lung (3.33 ± 0.29 AU), and spleen (3.67 ± 0.24 AU) of mice injected with PBMCs, with scores tending to be lower in organs of mice co-injected with autologous human TX200-TR101 Tregs plus PBMCs compared to those injected with PBMCs only (intestine 0.45 ± 0.21 AU, liver 1.75 ± 0.22 AU, lung 2.5 ± 0.29 AU, and spleen 2.75 ± 033 AU) (Fig. 3B). In human skin grafts, CD45 scores were slightly higher in mice co-injected with autologous human TX200-TR101 Tregs and PBMCs compared with those injected with PBMCs only (1.25 ± 0.18 AU and 0.56 ± 0.24 AU, respectively) but not statistically significant (*p* = 0.08 per 2-way ANOVA). In mice injected with human TX200-TR101 Tregs only, human CD45 was only detected at low levels in the human skin grafts (CD45 score 0.167 ± 0.17 AU) and not in the other organs analyzed, indicating specificity for their target antigen HLA-A*02. Human FOXP3 immunopositivity was only detected in human skin grafts of mice co-injected with autologous human TX200-TR101 Tregs and PBMCs and in mice injected with human TX200-TR101 Tregs alone (FOXP3 scores of 1.75 ± 0.18 AU and 1.33 ± 0.33 AU, respectively). Quantification of CD45^+^ and FOXP3^+^ cells in the skin transplant harvested at day 28 from mice injected with PBMCs plus TX200-TR101 or with TX200-TR101 only showed that 61.4 ± 4.59% (mean ± SEM; 7 field views; 3 mice) and 75.9 ± 7.29% (mean ± SEM; 6 field views; 2 mice) of CD45^+^ cells were also positive for FOXP3, respectively.

No significant decrease in skin graft histology scores performed at day 28 was observed in mice co-injected with autologous human TX200-TR101 Tregs and PBMCs compared with those injected with PBMCs only (10.6 ± 0.88 [*n* = 8] versus 13.2 ± 0.86 [*n* = 9], respectively [mean ± SEM]). At day 35, a slight increase in skin graft histology score was observed in animals receiving PBMCs plus TX200-TR101 Tregs (14.75 ± 1.18, *n* = 4) and in animals receiving TX200-TR101 Tregs alone (4.50 ± 0.50 at day 28 [*n* = 2] versus 13.0 ± 1.73 at day 35 [*n* = 3]) (data per mouse and means for the pooled data are presented in Fig. 3C). However, skin graft rejection was delayed in animals receiving PBMC + TX200-TR101 or TX200-TR101 alone compared to PBMCs alone with a median survival of 35 days for the PBMC + TX200-TR101 and TX200-TR101 alone groups versus 16 days for PBMCs alone (Fig. 3D).

### No transformation of TX200-TR101 Tregs in vitro and senescence following chronic stimulation

**Fig. 4:**
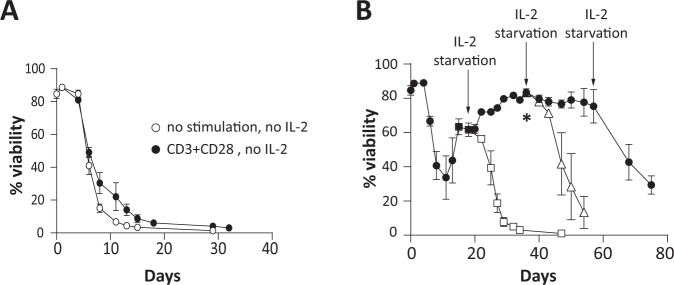
Expansion protocol does not induce the growth of IL-2-independent Treg clones. TX200-TR101 Tregs were cultured for up to 75 days either unstimulated or with TCR chronically stimulated with anti-CD3/CD28-coated dynabeads, in the presence or absence of high-dose IL-2 (1000 U/ml). 3 Treg donors were used with an average CAR transduction of 47 ± 3.0% (mean ± SEM) (**A**) Percent viability of TX200-TR101 Tregs over time, cultured without IL-2 and with or without TCR stimulation with anti-CD3/CD28-coated dynabeads. **B** Percent viability of TX200-TR101 Tregs over time, cultured with high-dose IL-2 without TCR stimulation. Arrows indicate IL-2 removal (starvation) and asterisk indicates reduction of IL-2 from 1000 IU/ml to 300 IU/ml. Black circles: viability of Tregs cultured with high dose of IL-2; white squares: viability of Tregs after first IL-2 removal; white triangles: viability of Tregs after second IL-2 removal. Data are presented as mean ± SEM (*n* = 3 Treg batches).

**Fig. 5:**
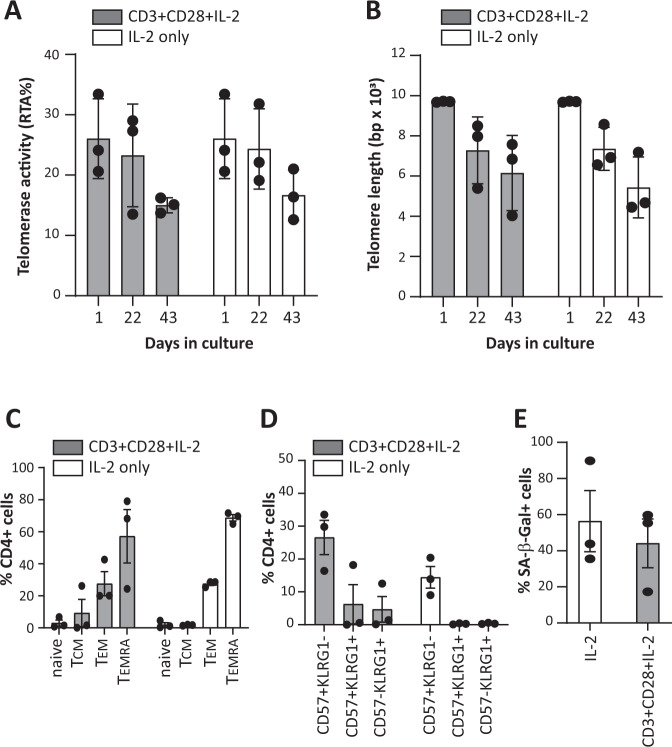
Expanded HLA-A*02-specific CAR-Tregs show signs of senescence after extended in vitro culture. TX200-TR101 Tregs were cultured with high-dose IL-2 (1000 U/ml), either unstimulated or with TCR chronically stimulated with anti-CD3/CD28-coated dynabeads. 3 Treg donors were used with an average CAR transduction of 47 ± 3.0% (mean ± SEM) (**A**) Telomerase activity after 1, 22, and 43 days in culture, presented as mean % RTA ± SD. **B** Telomere length after 1, 22, and 43 days in culture, presented as mean telomere length (bp) ± SD. **C** Phenotypic analysis of sub-populations of CD4^+^ Tregs after 54 days of culture with IL-2 only or with anti-CD3/CD28-coated dynabeads plus IL-2 (means ± SEM). **D** Expression analysis of CD57 and/or KLRG1 cell surface markers on CD4^+^ Tregs after 54 days culture with IL-2 only or with anti-CD3/CD28-coated dynabeads plus IL-2 (means ± SEM). **E** Expression of SA-β-Gal activity in CD4^+^ Tregs after 54 days of culture with IL-2 only or with anti-CD3/CD28-coated dynabeads plus IL-2 (means ± SEM). Bars represent mean ± SD/SEM and each dot represents a Treg batch (*n* = 3 Treg batches, 5 technical replicates were assessed per batch).

As no relevant in vivo tumorigenicity model is available for Tregs, TX200-TR101 Tregs were tested in vitro to assess whether transduction with the lentiviral vector TX200 or the cell culture process induced cellular or molecular alterations increasing the risk of tumorigenicity. When cultured in the absence of IL-2, the viability of TX200-TR101 Tregs decreased rapidly, with <10% of viable cells after 2 weeks, and no viable cells remaining after 1 month in culture (Fig. 4A). Similar results were observed during chronic TCR stimulation with anti-CD3/CD28 dynabeads. In the presence of high-dose IL-2, viability of TX200-TR101 Tregs was maintained up to 75 days but removal of IL-2 still induced cell death (Fig. 4B). Accordingly, TX200-TR101 Tregs were dependent on IL-2 for survival and cell growth, irrespective of chronic TCR stimulation. Relative telomerase activity (RTA) decreased over time in TX200-TR101 Tregs cultured in the presence of high-dose IL-2 and anti-CD3/CD28 dynabeads (from mean ± SD 26.0 ± 3.8% RTA at day 1 to 15.0 ± 0.7% RTA at day 43 for cells stimulated with anti-CD3/CD28-coated dynabeads plus IL-2 and 16.7 ± 2.4% RTA for cells cultured with IL-2 only) as did telomere length (from mean ± SD 9699.0 ± 14.4 base-pairs [bp] to 6154.3 ± 1079.8 bp at day 43 for cells stimulated with anti-CD3/CD28-coated dynabeads plus IL-2 and 5438 ± 874.7 bp for cells cultured with IL-2 only) (Fig. 5A, B), suggestive of senescence despite IL-2 and TCR stimulation. Senescence was also demonstrated after 54 days of culture with IL-2 by analysis of other senescence markers. Specifically, subpopulations of CD4^+^ Tregs were analyzed after 54 days in culture using CD45RA and CD27 to distinguish naïve, T central memory (T_CM_), T-effector memory (T_EM_) and terminally differentiated effector memory (T_EMRA_) cells (Fig. 5C). Tregs cultured in the presence of IL-2 alone or with anti-CD3/CD28-coated dynabeads plus IL-2 displayed mostly a T_EM_ phenotype (mean ± SD 27.5 ± 1.8% and 27.5 ± 13.0% on average for IL-2 or anti-CD3/CD28-coated dynabeads plus IL-2 culture conditions, respectively) or a T_EMRA_ phenotype (68.7 ± 3.4% and 57.2 ± 28.9% on average for IL-2 or anti-CD3/CD28-coated dynabeads plus IL-2 cultures, respectively). Flow cytometric analysis of expression of CD57 and killer cell lectin-like receptor G1 (KLRG1), two markers of senescence, showed that 14.4 ± 5.7% (mean ± SD) of CD4^+^ Tregs expressed CD57 when cultured with IL-2 only whilst 26.6 ± 9% expressed it when cultured with anti-CD3/CD28-coated dynabeads plus IL-2. Almost no expression of KLRG1 was detected except on cells cultured with anti-CD3/CD28-coated dynabeads plus IL-2 (10.9 ± 8.6% of CD4^+^ Tregs) (Fig. 5D). Senescence acidic β-galactosidase activity (SA-β-Gal) was also analyzed after 54 days in culture and was detected in 56.4 ± 29.2% of Tregs cultured with IL-2 only and in 44.1 ± 23.3% of Tregs cultured with anti-CD3/CD28-coated dynabeads plus IL-2 (Fig. 5E).

### Quality assessment of TX200-TR101 Tregs

**Fig. 6:**
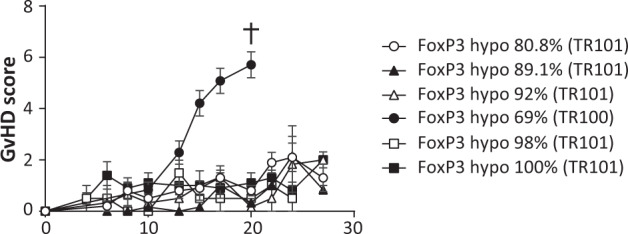
In vivo effects of different hypomethylation levels on GvHD scores. Male and female 8- to 11-week old NSG HLA-A*02 mice were injected intravenously with different batches of TX200 Tregs that were isolated with 2 different gating strategies (TR101 and TR100) and characterized by different levels of *FOXP3* hypomethylation. Mice were observed for up to 27 days or until ethical endpoints were reached (represented by a cross). Evolution of GvHD scores is shown based on scoring in 5 categories (0: no findings, 1: mild, 2: moderate, 3: severe) combined into a total score. Data are presented as mean ± SEM with *n* = 5 for 80.8% hypomethylation, *n* = 3 for 92% and 89.1%, *n* = 20 for 69%, *n* = 2 for 98%, and *n* = 5 for 100%. Average percentage of transduction was 59.5 ± 2.7% (mean ± SEM) and cells were on average 95.3 ± 2.8% CD4^+^/CD45RA^+^/CD25^+^/FoxP3^+^ (mean ± SEM).

Any contaminant Tconvs transduced with the CAR could potentially become proinflammatory effector T cells upon activation via the HLA-A*02 antigen and produce inflammatory cytokines, leading to cytotoxic effects. For safety purposes, it is therefore essential that the cell population used is depleted of Tconv. Flow cytometric T-cell phenotyping of TX200-TR101 demonstrated that only trace levels of contaminant non-Treg T cells were included (see supplementary, Table S4 and representative FACS plot Fig. S6). Epigenetically, Tregs and Tconvs can be differentiated according to hypomethylation of the *FOXP3* TSDR, which is fully demethylated in Tregs, whereas Tconv transduced with the CAR might transiently express FOXP3 upon activation but the *FOXP3* locus would not be demethylated [25, 26]. Therefore, low level of hypomethylation of the *FOXP3* locus of TX200-TR101 Treg preparation may indicate some contamination by Tconv. In early studies, HLA-A*02 CAR-Tregs (TR100) were isolated with a less stringent gating strategy in which the gate was set first on all CD25^+^/CD127^low^ cells (rather than on CD25^high^ cells) followed by gating on CD45RA^+^ cells, including some CD45RA^int^ cells. These cells had a *FOXP3* TSDR hypomethylation level of only 69% and caused rapid body weight reduction and GvHD, such that mice had to be euthanized starting less than 2 weeks post-injection and none survived longer than 19 days. In contrast, with the TX200-TR101 gating strategy, CAR-Tregs had a minimum level of 80.8% hypomethylation (Fig. 6) and no signs of GvHD were observed in mice injected with TX200-TR101 CAR-Tregs over 4 weeks (27 days) post-injection.

### Engrafted TX200-TR101 Tregs are stable and safe in a humanized HLA-A*02 NSG mouse model

Biodistribution, persistence, and potential toxicity of TX200-TR101 were analyzed over a 3-month period in humanized HLA-A*02 NSG mice, expressing the target antigen HLA-A*02 in all tissues [21]. TX200-TR101 was well tolerated by HLA-A*02 NSG mice, with no signs of clinical toxicity and no effect on group body weight nor signs of GvHD (no GvHD score >3) over the course of the study, with the exception of one mouse; this mouse had a score of 6 at day 47 post-injection, accompanied by weight loss up to 17%, and was found dead at day 50. One further mouse was euthanized at day 35 due to a tumor of ~0.9 mm^3^ on its left flank, categorized histopathologically as a highly vascularized soft-tissue osteosarcoma, likely of mouse origin. Flow cytometry revealed a low-level HLA-A*02 CAR-Treg infiltrate in the unperfused tumor (3 × 10^4^ cells in 0.9 mm^3^, suggesting that this tumor did not result from Treg activity). With 1 mouse lost prematurely due to onset of GvHD, overall survival at the scheduled termination was 95.8% (23/24). Scheduled culls at months 2 and 3 included 7 mice each. No macroscopic signs of tumorigenicity were observed in any of the organs analyzed at scheduled necroscopy. Histopathology revealed spleen hypercellularity and extramedullary hematopoiesis and congestion of lung, with infiltration of lymphocytes around pulmonary vessels, whilst no microscopic abnormalities were observed in the kidney, liver, or ovaries. No structural abnormalities were detected in testis, but there was no spermatogenesis. As busulfan is a known inhibitor of spermatogenesis in mice and rats [27, 28], the observed inhibition of spermatogenesis in the male mice is likely related to conditioning with busulfan, rather than effects of TX200-TR101. No TX200-TR101 engraftment-related toxicity or other tissue abnormalities were observed.

**Fig. 7:**
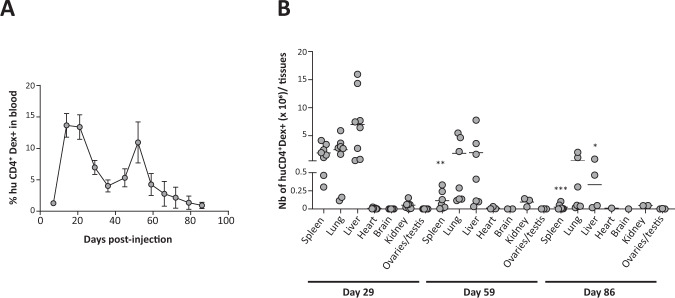
In vivo biodistribution study. Male and female 8- to 10-week old, humanized HLA-A*02 NSG mice (*n* = 24) were injected intravenously with TX200-TR101 Tregs. The percentage of transduction of Tregs was 49%. Mice were observed up to 3 months, with planned sacrifice after 1 month (day 29, *n* = 8), 2 months (day 59, *n* = 7), and 3 months (day 86, *n* = 7) (2 unscheduled deaths occurred). **A** Percentage of human HLA-A*02 CAR-Tregs (CD4^+^ HLA-A*02 dextramer [Dex]^+^) cells in blood over time. Data are presented as mean ± SEM. **B** Number of human HLA-A*02 CAR-Tregs (CD4^+^Dex^+^) cells in different tissues based on flow cytometry. Data are presented as scatter plots, with each dot representing a mouse and lines representing the mean. Asterisks indicate statistical significance compared with day 29, with *p < 0.05 and ***p < 0.001 calculated using Kruskal-Wallis multiple comparison test with Dunn’s post-hoc test (*n* = 24 until day 29, *n* = 16 until day 31, *n* = 15 until day 50, *n* = 14 until day 59 and *n* = 7 thereafter).

Multicolor flow cytometry was used to assess the expansion efficiency and phenotype of TX200-TR101. Human HLA-A*02 CAR-Tregs (CD4^+^HLA-A*02 dextramer^+^) in blood decreased over time but were still present at month 3 (from mean ± SEM 13.4 ± 1.96% human CD4^+^ HLA-A*02 dextramer^+^ cells 3 weeks post-injection to 4.26 ± 1.76% at month 2 and 0.93 ± 0.49% at month 3) (Fig. 7A). The highest levels of human HLA-A*02 CAR-Tregs in tissues were detected in spleen, lung, and liver, whilst only low levels were found in heart, brain, kidney, and reproductive organs (Fig. 7B). Human HLA-A*02 CAR-Tregs in tissues decreased over time, but were still present at month 3, demonstrating persistence over 3 months. All human HLA-A*02 CAR-Tregs in spleen, lung, and liver expressed FOXP3 at month 1 (mean ± SEM 99.8 ± 0.08% FOXP3^+^ in spleen, 98.7 ± 0.68% FOXP3^+^ in lung, and 99.5 ± 0.22% FOXP3^+^ in liver) and high percentages were still observed at month 2 (80.1 ± 3.91% FOXP3^+^ in spleen, 75.8 ± 3.18% FOXP3^+^ in lung, and 77.3 ± 3.62% FOXP3^+^ in liver), while decreased FOXP3 levels were observed at month 3 in liver (99.5 ± 0.22% FOXP3^+^ in spleen, 77.3 ± 3.61% FOXP3^+^ in lung, and 56.4 ± 4.0% FOXP3^+^ in liver), indicating a stable phenotype of TX200-TR101 Tregs for up to 2 months (data not shown).

**Fig. 8:**
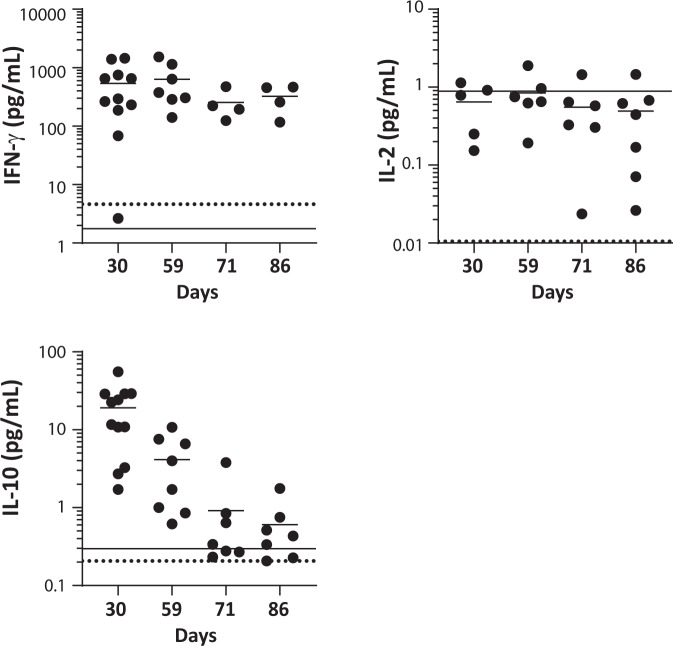
Evaluation of cytokine production in HLA-A*02 NSG mice receiving HLA-A*02 CAR Tregs. Male and female 8- to 10-week old, humanized HLA-A*02 NSG mice (*n* = 24) were injected intravenously with TX200-TR101 Tregs. Mice were observed up to 3 months, with planned sacrifice after 1 month (day 29, *n* = 8), 2 months (day 59, *n* = 7), and 3 months (day 86, *n* = 7) (2 unscheduled deaths occurred). Plasma human cytokines were measured by multiplex immunoassay in peripheral blood at days 30, 59, 71, and 86 post-administration of TX200-TR101. IL-13, IL-1β, IL-12p70, IL-6, and IL-17A/F could not be detected in any mice (below quantification level). Data are presented as scatter plots, with each dot representing a mouse and lines representing the mean. Dotted lines indicate level of cytokine in naïve HLA-A*02 NSG mice (merged with the *x*-axis if close to zero) and the plain line represents the lower limit of quantification.

Plasma human cytokine levels did not indicate a switch of the injected human Tregs to a proinflammatory phenotype (Fig. 8). No increased levels of proinflammatory cytokines were observed compared to naïve HLA-A*02 NSG mice; the levels of IL-6, IL-12p70, IL-1β, IL-13 and IL-17A/F were below the lower limit of quantification. Very low levels of IL-2 were detected and did not increase over time (mean ± SEM 0.65 ± 0.19 pg/ml at day 30 to 0.49 ± 0.19 pg/ml at day 86). Levels of IFNγ were increased, from 540.8 ± 150.7 pg/ml 30 days post-administration to 323.7 ± 83.7 pg/ml 86 days post-administration. This increase in IFNγ levels was consistent with the production of this cytokine in vitro by TX200-TR101 Tregs activated through the CAR using HLA-A*02 dextramer measured in culture supernatant (171.1 ± 40.1 pg/ml, *n* = 12), with levels considered low compared to the level of IFNγ produced by lots contaminated with CAR-T-effector cells (12375 ± 7890 pg/ml, *n* = 5). The anti-inflammatory and immunomodulatory cytokine IL-10 was increased at day 30 post-administration of TX200-TR101 and decreased over time (19.1 ± 4.49 pg/ml at day 30 to 0.60 ± 0.20 at day 86).

### Engrafted TX200-TR101 Tregs are not significantly impaired by tacrolimus treatment

**Fig. 9:**
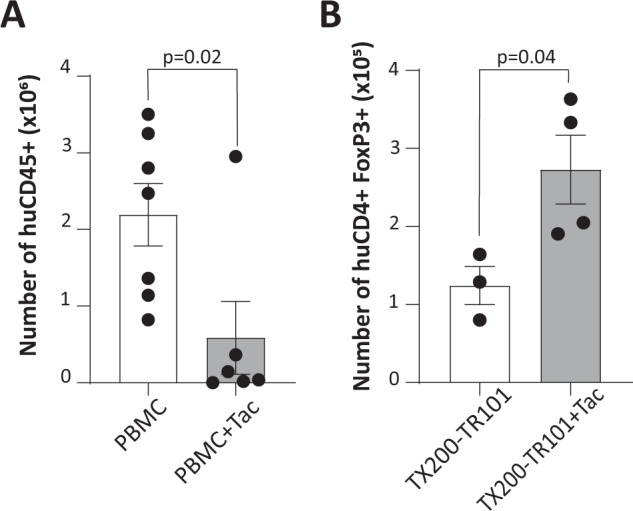
Interaction with tacrolimus in vivo. Female, 7-week old HLA-A*02 NSG mice (*n* = 20 total) were injected intravenously with either human PBMCs or with TX200-TR101 Tregs. The percentage of transduction of Tregs was 54.6%. Starting from 7 days post-cell administration, some groups received intraperitoneal injections of 0.5 mg/kg tacrolimus (Tac), administered daily for 2 weeks. **A** Number of human CD45^+^ cells in spleen 3-weeks post-PBMC injection, assessed by flow cytometry. Data are presented as scatter plots with each dot representing a mouse, bars representing the mean, and error bars SEM (*n* = 7 for PBMC and *n* = 6 for PBMC plus Tac). Statistical significance between groups was determined per time point using t-test. **B** Number of human CD4^+^ FOXP3^+^ Tregs in spleen after 3 weeks, assessed by flow cytometry. Data are presented as a scatter plots, with each dot representing a mouse, bars representing the mean, and error bars SEM (*n* = 3 for TX200-TR101 Tregs and *n* = 4 for TX200-TR101 Tregs plus Tac). Statistical significance between groups was determined using t-test.

Tacrolimus is a standard-of-care immunosuppressant in solid organ transplantation, described as deleterious to the survival of Tregs [29]. It was therefore tested whether the engraftment of CAR-Tregs and their phenotype in the HLA-A*02 NSG mouse model would be maintained in the presence of tacrolimus. In a 2-week pilot study using tacrolimus doses ranging from 0.1 to 2 mg/kg/day, doses over 0.5 mg/kg/day resulted in weight loss >20% and GvHD-like symptoms in mice (not shown). It has been described that tacrolimus induces hypertension and has a nephrotoxic effect in mice [30, 31]. Mice that received PBMCs plus tacrolimus (0.5 mg/kg/day) showed a statistically significant reduction in the number of PBMCs found in the spleen in comparison to mice that received PBMCs only (mean ± SEM 5.87 × 10^5^ ± 4.76 × 10^5^ human CD45^+^ cells versus 2.19 × 10^6^ ± 4.07 × 10^5^ human CD45^+^ cells, respectively) (Fig. 9A) indicating that this dose was sufficient to reduce PBMC engraftment. In mice that received TX200-TR101 Tregs, the engraftment of cells in spleen was not impaired and was significantly increased in mice injected with tacrolimus (1.24 × 10^5^ ± 2.44 × 10^4^ CD4^+^FOXP3^+^ cells versus 2.73 × 10^5^ ± 4.40 × 10^4^ CD4^+^FOXP3^+^, respectively) (Fig. 9B). No effect of tacrolimus was seen on FOXP3 expression in CD4^+^ HLA-A*02-negative cells (i.e. TX200-TR101 Tregs), with 93.5% of CD4^+^ cells expressing FOXP3 in the spleen in untreated mice compared to over 94% of CD4^+^ cells expressing FOXP3 after 2-week treatment, indicating no impairment of Treg stability (not shown).

## Discussion

Functionality of TX200-TR101 Tregs could be shown in vitro and in vivo, demonstrating efficacy of the clinical candidate to induce immunosuppression, in line with the results observed with a previous proof-of-concept study [8]. In vitro, TX200-TR101 Tregs could be activated specifically by their target antigen HLA-A*02 and were able to suppress Tconv proliferation once activated. When co-cultured with characterized human PBMCs, TX200-TR101 Tregs were activated by all tested HLA-A*02 alleles and showed no cross-reactivity with other tested alleles, demonstrating specificity towards the HLA-A*02 antigen. The lack of activation by HLA-A*02-negative PBMCs also indirectly implies that there was no cross-reactivity with other HLA subtypes expressed by these cells. The proof-of-concept study observed binding of the ScFv from which the clinical candidate was derived to HLA-A*68, HLA-A*25, and HLA-A*69 in a bead assay [8]. However, it was also shown that the binding to these antigens was largely reduced compared to the original mouse HLA-A*02 ScFv (BB7.2). In our experiments, TX200-TR101 Tregs showed no relevant cellular activation by HLA-A*68 or HLA-A*25 (HLA-A*69 PBMCs could not be obtained as the haplotype is very rare) suggesting that the residual binding observed in a bead assay is not sufficient to induce the activation of the cells. As cross-reactivity to HLA-A*69 could not be assessed, subjects carrying this specific HLA-A allele will be excluded from the clinical trial.Humanized mouse models are well established tools to study human Treg function in vivo and have been used to successfully investigate their role in prevention of xenogeneic GvHD [32] and rejection of various xenografts such as skin, islets, and arteries [33]. In the xenogeneic GvHD model, TX200-TR101 Tregs reduced proinflammatory effector T cell activity, as shown by prevention of the engraftment, expansion, and activity of cytotoxic and proinflammatory T cells and thus prevented GvHD onset. It was demonstrated that TX200-TR101 Tregs were more potent at preventing GvHD than polyclonal Tregs as it was previously shown in other published studies (by [8, 10] and more recently by [11, 34]).

A human skin transplant model was also analyzed, due to its technical feasibility, the possibility to transplant human material, and for its comparability to other published studies using HLA-A*02 CAR-Tregs [7–9]. Although TX200-TR101 Tregs were minimally effective in preventing transplant rejection, they specifically localized to the HLA-A*02-positive human skin graft. It has been shown that it is notoriously difficult to reduce skin graft rejection in NSG mice. In NSG mice, human skin transplants develop an inflammatory infiltrate consisting predominately of host Gr1^+^ cells that is detrimental to the survival of human endothelium in the graft [35]. In fact, we observed that the human skin grafts on mice that did not receive PBMCs showed microscopic signs of fibrosis, mixed leukocyte infiltration consisting primarily of macrophages, inflammation and proliferation in the dermis, and hyperkeratosis of the epidermis. These findings were exacerbated in mice that received PBMCs. This infiltration of Gr1^+^ cells and damage to the skin graft caused by these cells could not be reversed by TX200-TR101, hence the minimal effect seen on the graft rejection score. For the mice that received TX200-TR101 alone, skin graft histology score was also increased at day 35. It was observed that in this group, skin grafts showed some increased infiltration by neutrophils and macrophages and signs of inflammation. This phenomenon has been described in a publication from Moreau et al., where it was reported that Tregs promote innate inflammation after skin barrier breach via transforming growth factor-β activation [36]. This publication also showed that Tregs in skin could attract neutrophils towards injured skin and thus delay epidermal regeneration. However, it was observed that skin graft rejection was delayed in mice receiving TX200-TR101.The findings were not related to poor functional capacity of the TX200-TR101 Tregs used in these experiments as they effectively prevented GvHD onset in NSG mice.

Biodistribution and safety of the clinical candidate were assessed in a variety of in vitro and in vivo studies, of which the most relevant are presented in this publication. Whilst no appropriate in vivo tumorigenicity model is available, in vitro experiments did not show evidence for tumorigenicity. TX200-TR101 Tregs depended on IL-2 for survival and growth and became senescent during chronic stimulation. Only trace levels of contaminant non-Treg CAR-T cells were found in TX200-TR101 preparations, indicating a favorable ratio of Tregs over Tconvs. *FOXP3* TSDR hypomethylation can identify Tregs [25, 26]. We found cells with a minimum of 80% TSDR hypomethylation were safe and efficacious in GvHD prevention. First-generation TX200-TR100 Tregs using a previous sorting strategy had low hypomethylation and caused rapid GvHD onset.

No major safety concerns arose in a 3-month safety and bioavailability study following a single intravenous administration of TX200-TR101 in humanized HLA-A*02 NSG mice. Only one of 24 analyzed mice developed GvHD and only one tumor was observed. The tumor was an osteosarcoma, probably of mouse origin, with a low level of CAR-Treg infiltrate. The infiltrate might have been circulating CAR-Tregs, as the tumor was highly vascularized and not perfused before harvesting. Of note, a longer study duration would have been beneficial for tumorigenicity assessment, but it was not expected initially that TX200-TR101 would have such a long persistence in the absence of human IL-2.

The distribution of human HLA-A*02 CAR-Tregs cells in the humanized HLA-A*02 NSG mice was consistent with literature describing that intravenous injection of cells in mice results in their capture in the pulmonary vasculature, due to the anatomical localization of the lung as the first capillary bed post intravenous injection, with cells redistributing later mainly to well vascularized organs such as the spleen, liver, and kidney [37–39]. TX200-TR101 infiltrated mainly the lung, as first point of entry from blood, then spleen and liver, and engaged with their target antigen HLA-A*02 that resulted in their activation and proliferation. Histopathology showed congestion and infiltration of lung, also consistent with the injection by intravenous route and with the engagement and retention of HLA-A*02 CAR-Tregs by interaction with HLA-A*02 expressed on lung endothelium. Histopathologic findings also indicated extramedullary hematopoiesis in the spleen, likely due to expansion of TX200-TR101 Tregs following recognition of splenic HLA-A*02. Reassuringly, only very few TX200-TR101 Tregs were found in the heart, brain, and reproductive tract.

Treg senescence was assessed in an in vitro model consisting of chronic stimulation via their TCR in the presence of high-dose IL-2. This assay showed that constant Treg stimulation induced the increase of T_EMRA_ cells. After 54 days in culture, CD4+ Tregs expressed low levels of the senescence markers CD57 and KLRG1 whereas acidic β-galactosidase activity (SA-β-Gal) was increased. CD57 and KLRG1 are commonly used as senescence markers in CD4+ and CD8+ T cells to assess T cells fitness after Ebola vaccination [40], in cancer [41] or autoimmunity [42], but their relevance in Tregs is not well defined. However, the SA-β-Gal marker was used in a model of aging mice to assess the senescence status of Tregs [43]. In the biodistribution study, 3 months post-administration of TX200-TR101, the number of cells recovered in tissues was too low to reliably perform the SA-β-Gal test or analyze the level of CD57 and KLRG1 expression. Due to these limitations, more work will be required to validate whether these markers could be used to assess the senescence status of CAR Tregs in humanized mouse models.

One concern in Treg immunotherapy is a lack of Treg stability and potential conversion into a pro-inflammatory phenotype [3]. In the 3-month in vivo study, TX200-TR101 Tregs demonstrated in vivo persistence and a stable phenotype in terms of FOXP3 expression and a lack of proinflammatory cytokine production. As Tregs rely on IL-2 [44], it was unexpected that TX200-TR101 Tregs persisted up to month 3 in the mice without IL-2 administration, demonstrating they only require a low level of IL-2 to survive. Of note, injection of TX200-TR101 in NSG mice devoid of the HLA-A*02 transgene did not result in cell engraftment. In this setting, cells rapidly disappeared in blood and spleen, indicating that they require CAR-mediated signaling to survive in the absence of IL-2 (data not shown). More work would be required to understand which type of signals the CAR-Tregs receive in vivo to survive.

Noteworthy, our planned Phase 1/2 trial is expected to be the first to evaluate CAR-Tregs in humans. In clinical trials with polyclonal and non-genetically-modified, antigen-specific Tregs, no severe or life-threatening acute toxicities, nor increases in the number of opportunistic infections or de-novo malignancies have been reported to date [4, 5]. The recently published ONE Study evaluated 28 kidney transplant recipients treated with polyclonal (*n* = 23) or donor-reactive (*n* = 5) Tregs, compared with 70 recipients receiving standard immunosuppressive therapy [5]. Combined adverse event and acute rejection data in the ONE Study revealed no safety concerns. No differences in biopsy-confirmed acute rejection rates were observed between Treg and conventional therapy groups – notably using mostly polyclonal non-allospecific Tregs that are expected to have a considerably lower efficacy compared to antigen-targeted Tregs. One of the individual trials analyzed within the ONE Study found a trend towards a reduced rejection rate in the Treg group compared to the reference group [45]. Of note, 40% of patients receiving cell-based therapies in the ONE Study could taper immunosuppressive treatments to monotherapy whilst most patients in the reference group continued at least on dual immunosuppression, and a lower rate of infections was observed with cell-based therapies [5].

In oncology settings, CAR-modified T cell therapies using autologous Tconv have shown significant acute toxicities, mostly related to cytokine release syndrome, occurring when large numbers of CAR-Tconv are activated to release a variety of proinflammatory cytokines and chemokines [6]. Cytokine release syndrome has not been described to date in studies utilizing polyclonal or antigen-specific Treg therapies [4, 5], likely explained by the differing mechanism of action of Tregs that inhibit inflammatory responses by releasing immunomodulatory cytokines in contrast to the proinflammatory properties of Tconv. Quality assessments of our clinical candidate TX200-TR101 have detected only trace levels of Tconv in the cell product and strict release criteria will ensure a favorable ratio of Tregs over Tconv to minimize the risk of Tconv transduction, thereby further reducing the risk of strong immune activation and proinflammatory cytokine release.

Tacrolimus is a standard-of-care immunosuppressant in solid organ transplantation [29]. It inhibits T cell proliferation in response to ligation of the TCR, impairs T-cell-mediated cytotoxicity, and has been described as deleterious to the survival of Tregs. Whilst showing some toxicity with GvHD-like symptoms in a GvHD mouse model, tacrolimus did not impair survival of TX200-TR101 Tregs. Surprisingly, tacrolimus promoted Treg engraftment in vivo. Such an effect on the Treg population had been previously observed in patients with atopic dermatitis treated with low dose of cyclosporin A, another calcineurin inhibitor [46]. These patients showed a significant increase of the CD4^+^/CD25^+^/CD127^low^ Treg population with similar suppressive activity in comparison to non-treated patients. It was concluded that there was no significant risk of drug interaction that would preclude using tacrolimus in the planned Phase 1/2 trial. Tacrolimus will be administered to all patients in the planned trial as standard of care.

In conclusion, preclinical experiments have shown that TX200-TR101 is specific, stable, efficacious, and safe, and its function is not expected to be impaired by concomitant tacrolimus treatment. These data, supported by clinical experience with other experimental Treg therapies, provided the basis for a Clinical Trial Application for the first-in-human study of TX200-TR101 in mismatched-donor renal transplant recipients (STEADFAST study, EudraCT 2019-001730-34). This open-label, single ascending dose, dose-ranging, Phase 1/2a study in HLA-A*02-negative subjects awaiting the receipt of a kidney transplant from an HLA-A*02-positive donor was initiated in 2021.

## Supplementary information

Supplementary data

## Data Availability

All data generated or analysed during this study are included in this published article [and its supplementary information files].
