# Supplementary material for: Preclinical assessment of antigen-specific chimeric antigen receptor regulatory T cells for use in solid organ transplantation
Source: Gene Ther. 2022 Aug 5;30(3-4):309–22. doi: 10.1038/s41434-022-00358-x (PMC10113151; doi:10.1038/s41434-022-00358-x)
Supplement: Supplementary file 1 — Supplementary data [file 41434_2022_358_MOESM1_ESM.pdf]

SUPPLEMENTARY

Preclinical assessment of antigen-specific chimeric antigen receptor regulatory T cells for use in solid organ transplantation

List of figures

Fig. S1: CAR constructs TX200 and TX235. 2
Fig. S2: Gating strategy for isolation of naïve Tregs. 4
Fig. S3: Flow cytometric analysis of human cell expansion in NSG mice in the GvHD experiment 5
Fig. S4: Gating strategy for assessment of human cell expansion in mice. 6
Fig. S5: Body weight changes post-delivery of PBMCs ± HLA-A*02 CAR Tregs (Batch 1 and 2) used in the skin transplant experiment. 9
Fig. S6: Gating strategy for assessment of the purity of TX200-TR101 batches. 11

List of tables

Table S1: Flow cytometry antibodies 3
Table S2: GvHD score in mouse models 7
Table S3: HLA Genotypes of PBMCs co-cultured with TX200-TR101 Tregs and percentage of TX200-TR101 Tregs expressing activation markers after co-culture 8
Table S4: Flow cytometric quantification of non-regulatory T cells in 3 batches of TX200-TR101 10

SUPPLEMENTARY MATERIALS AND METHODS

VECTOR DESIGN

Schematics of the lentiviral constructs for the human leukocyte antigen (HLA)-A*02-specific chimeric antigen receptor (CAR) TX200 and the control construct TX235 with non-signalling endodomain are shown in Fig. S1.

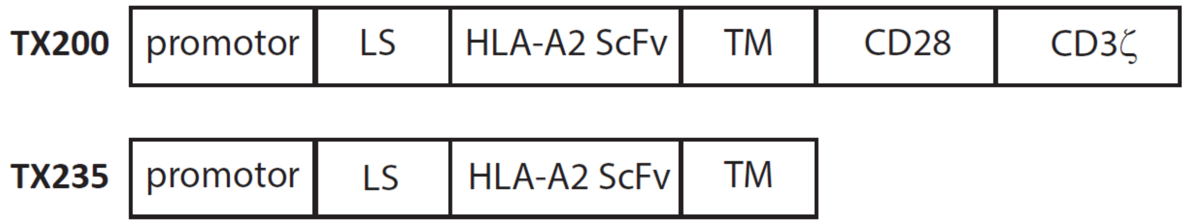

Fig. S1: CAR constructs TX200 and TX235.

Schematic of the HLA-A*02-specific CAR TX200 and the control construct TX235 with non-signalling endodomain. CD: cluster of differentiation; HLA-A*02 ScFv: CAR region specific for human HLA-A*02 single-chain variable fragment (ScFv); LS: lead sequence; TM: transmembrane domain.

FLOW CYTOMETRY

Details of antibodies used in flow-cytometric analyses are presented in Table S1.

Table S1: Flow cytometry antibodies

Antibody	Catalog number	Supplier
CD4 VioBlue	130-113-219	Miltenyi
CD69 APC Vio770	130-112-616	Miltenyi
GARP PE	130-103-819	Miltenyi
CD25 BV510	563352	BD Biosciences
Dextramer APC	WB2666-APC	Immudex
FOXP3	12-4777-42	ThermoFisher
FOXP3 AF647	560045	BD Biosciences
hCD15s BV421	562428	BD Biosciences
hCD25 BV510	563352	BD Biosciences
hCD39 SB600	63-0399-42	ebioscience
hCD4 FITC	557852	BD Biosciences
hCD4 FITC	555346	BD Biosciences
hCD4 PE-cy7	557852	BD Biosciences
hCD4 Percp-cy5.5	45-0049-42	eBioscience
hCD45 APC-cy7	557833	BD Biosciences
hCD45 VioGreen	130.110.638	Miltenyi
hCD8 BV421	562428	BD Biosciences
hCD8 SB702	67-0086-42	eBioscience
hCTLA-4 PE-cy7	369614	Biolegend
HLA-A2 BV421	740082	BD Biosciences
HLA-A2 PE	558570	BD Biosciences
huCD4	300554	Biolegend
huCD4 FITC	555346	BD Biosciences
huCD45	560777	BD Biosciences
huCD8	48-0087-42	ThermoFisher
IFN-ɣ FITC	554700	BD Biosciences
IFNg Vioblue	103-109-231	Miltenyi
IL-2 PE-Vio770	130-111-305	Miltenyi
Ki67 FITC	130-100-339	Miltenyi
mCD45	25-0451-82	ThermoFisher
mCD45 BV510	563891	BD Biosciences
mCD45 FITC	11-0451-82	eBioscience
mCD45APC Vio770	130-110-637	Miltenyi

The gating strategy for isolation of naïve regulatory T cells (Tregs) as cluster of differentiation (CD)4$^+$/CD45RA$^+$/CD25^{high}/CD127^{low} human cells is shown in Fig. S2. After gating on live lymphocytes and gating out doublets (not shown), human naïve CD4$^+$ cells were identified as CD4$^+$ and CD45RA$^+$ (purple square). Naïve Tregs were then identified as CD127^{low} and CD25^{high} as shown by the red gate.

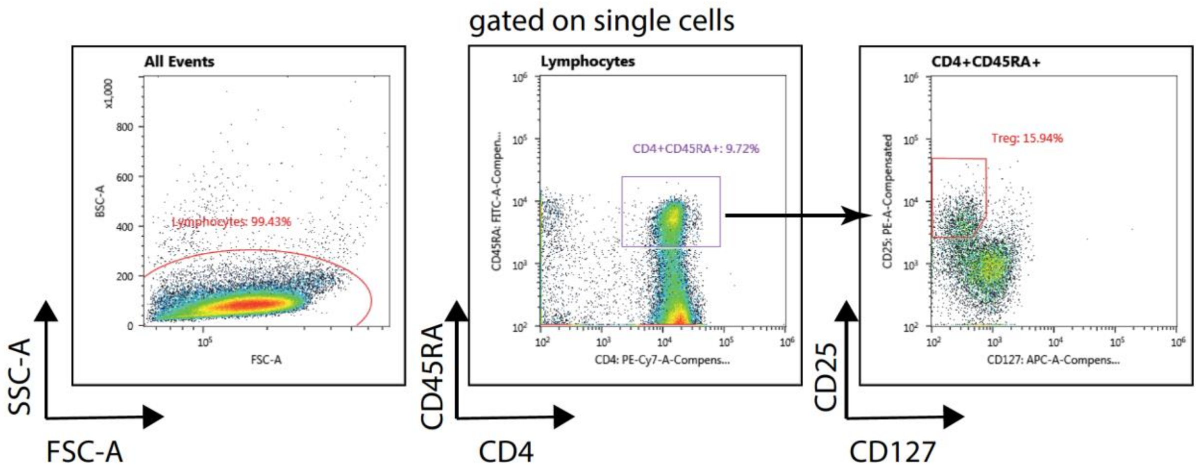

Fig. S2: Gating strategy for isolation of naïve Tregs.

Representative cytofluorimetric plots showing the gating strategy to define human naïve CD4$^+$ Tregs.

Representative FACS plots of human cells expansion in the blood of NOD *scid* gamma (NSG) mice obtained in the GvHD experiment are shown in Fig. S3. After gating on lymphocytes and gating out doublets (not shown), human cells were identified using human CD45 cell surface marker. Human CD45^{+} cells were then analysed for expression of CD4 and CD8 (not shown) and cell surface expression of HLA-A*02 to determine if the cells were derived from peripheral blood mononuclear cells (PBMCs) (HLA-A*02-positive) or from TX200-TR101 (HLA-A*02-negative).

This gating strategy was used in blood and tissues to estimate the percentages and number of cells of human origin that were CD45^{+}, CD4^{+}, CD8^{+} and the percentage and number of human CD4^{+} cells that express the HLA-A*02.

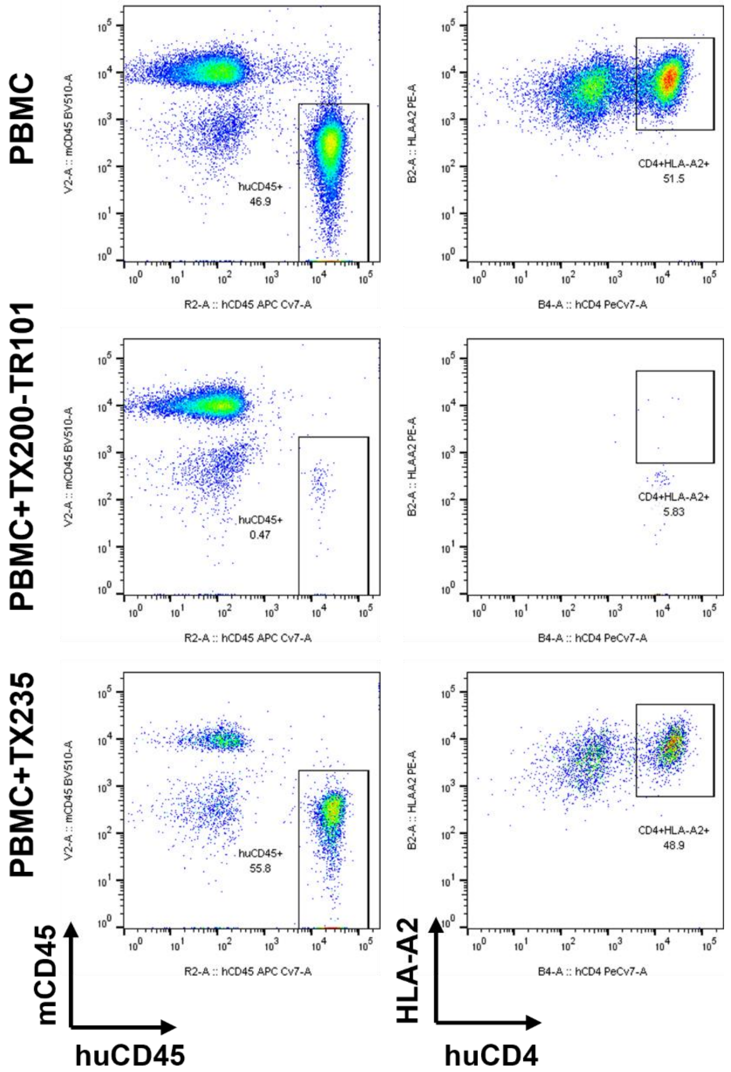

Fig. S3: Flow cytometric analysis of human cell expansion in NSG mice in the GvHD experiment

Representative cytofluorimetric plots showing the gating strategy to define human CD45+ and CD4+ HLA-A*02+ cells

An example gating strategy for assessment of human cell expansion in the blood of HLA-A*02 mice is shown in Fig. S4a. After gating on lymphocytes and gating out doublets, human cells were identified using human CD45 cell surface marker. Human CD45^{+} cells were then analysed for expression of CD4 and cell surface expression of dextramer was analysed on CD4^{+} cells.

The gate for dextramer-stained cells was set-up using the FMO control (cells stained with all surface markers except dextramer (Fig. S4b).

This gating strategy was used in blood and tissues to estimate the percentage of cells of human origin that were CD45$^+$, CD4$^+$, and the percentage of human CD4$^+$ cells that express the HLA-A*02 CAR (dextramer-positive).

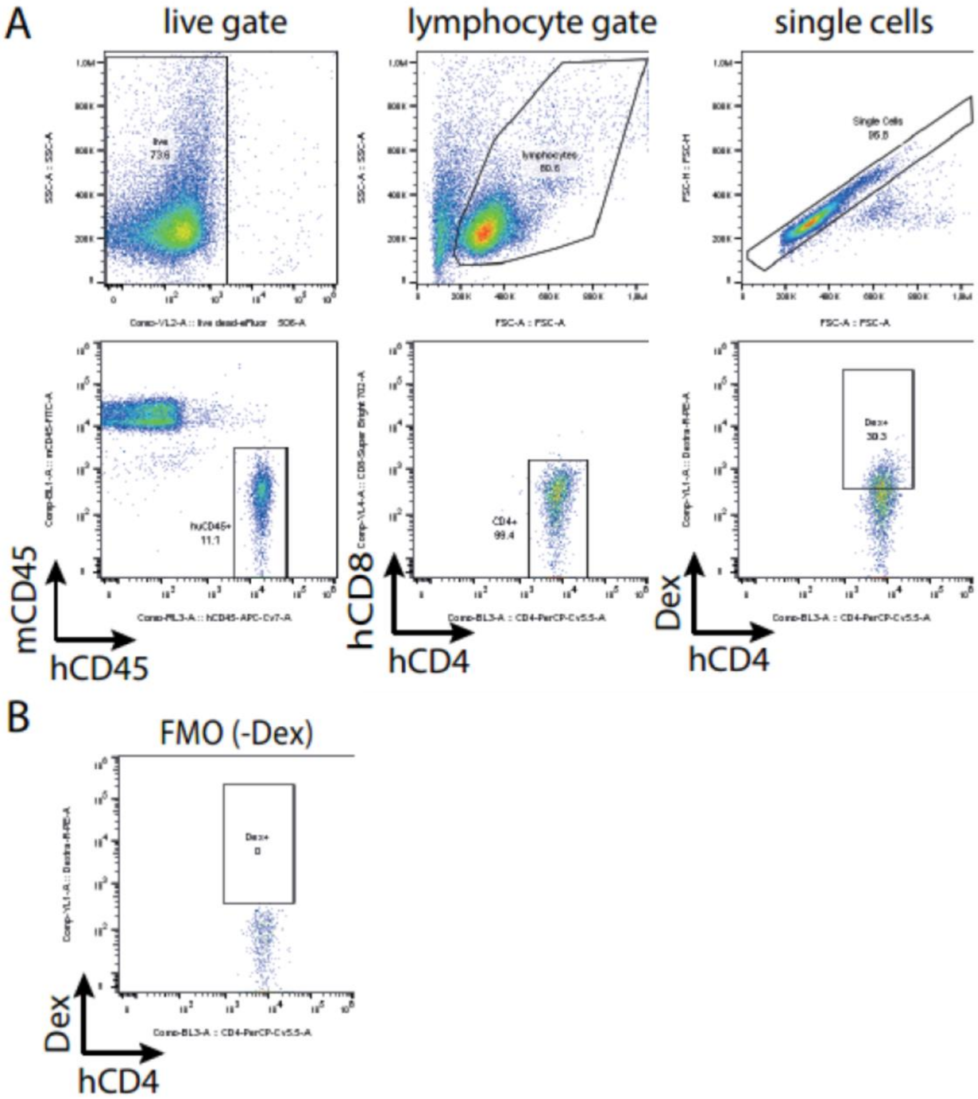

Fig. S4: Gating strategy for assessment of human cell expansion in mice.

a Representative cytofluorimetric plots showing the gating strategy to define human CD4$^+$ and CD8+ cells and CAR-Tregs using dextramer staining **b** FMO control plots used to set up the gates for dextramer-positive (Dex+) cells

MOUSE MODELS

Graft-versus-host disease (GvHD) was scored 3 times weekly based on weight, fur texture, posture, activity level, and skin integrity as detailed in Table S2. The action taken depending on the score is also included in this table. This scoring system was used in all in vivo experiments with the exception of the skin transplant model, where scoring was performed according to Cooke et al. 1996 [1].

Table S2: GvHD score in mouse models

	**Score**			
	**0**	**1**	**2**	**3**
**Weight loss**	<5%	5-10%	>10-15%	>15%
**Activity**	Mice move spontaneously and explore the cage (dig, eat, and drink)	Spontaneous activity is mildly reduced	Spontaneous activity is minimal but can be induced by gentle touch	Activity is seriously impaired. Mice do not move even if stimulated repetitively
**Hunch**	Not hunched	Mild hunched posture (round)	Moderate hunched posture (easily noticed)	Severe hunched posture (tip toe gait)
**Fur**	Normal	Ruffled	Fur loss (may be generalized with skin exposed by caressing or localized and presenting as alopecia)	Fur loss on 25% of mouse
**Skin**	Normal	Mildly inflamed (mildly red)	Moderately inflamed or affects face or footpads	Skin is ulcerated (face, footpads) NB: conjunctivitis is also a sign of GvHD (1=discharge, pus; 2=closing, whitening of cornea)
**Action**	None	None	Monitor mice every day[1]	Euthanize immediately

[1] Mice with a score of 2 in any category or a total score >4 were scored every day.

SUPPLEMENTARY RESULTS

IN VITRO CROSSREACTIVITY ASSAY

A summary of the HLA genotype of the PBMCs used in the cross-reactivity assay and expression of the activation markers CD69, CD71, and glycoprotein A repetitions predominant (GARP) in TX200-TR101 Tregs after co-culture is presented in Table S3 (green: HLA-A*02 positive PBMCs).

Table S3: HLA Genotypes of PBMCs co-cultured with TX200-TR101 Tregs and percentage of TX200-TR101 Tregs expressing activation markers after co-culture

HLA-A	HLA-B	HLA-C	HLA-DRB1	HLA-DQB1	HLA-DPB1	HLA-DQA1	HLA-DPA	CD69 (%)		CD71 (%)		GARP (%)	
								**Mean (n=3)**	*SD*	**Mean (n=3)**	*SD*	**Mean (n=3)**	*SD*
A*01:01 A*03:01	B*07:02 B*40:01	C*03:04 C*07:02	DRB1*04:04 DRB1*15:01	DQB1*03:02 DQB1*06:02	DPB1*04:01 DPB1*04:02	DQA1*03:02 DQA1*06:02	DPA1*01:03 DPA1*01:03	**5**	*1*	**4**	*3*	**5**	*2*
A*01:01 A*24:02	B*38:02 B*40:01	C*04:01 C*07:01	DRB1*11:04 DRB1*13:02	DQB1*03:01 DQB1*06:04	DPB1*04:01 DPB1*04:01	DQA1*01:02 DQA1*05:05	DPA1*01:03 DPA1*01:03	**9**	*4*	**6**	*2*	**9**	*4*
A*01:01 A*25:01	B*15:17 B*18:01	C*07:01 C*12:03	DRB1*13:03 DRB1*15:01	DQB1*03:01 DQB1*06:02	DPB1*04:01 DPB1*04:01	DQA1*01:02 DQA1*05:05	DPA1*01:03 DPA1*01:03	**8**	*2*	**6**	*4*	**7**	*3*
A*01:01 A*29:02	B*08:01 B*49:01	C*07:01 C*07:01	DRB1*04:07 DRB1*07:01	DQB1*02:02 DQB1*03:01	DPB1*04:01 DPB1*16:01	DQA1*02:01 DQA1*03:04	Not tested	**4**	*3*	**2**	*1*	**4**	*1*
A*01:01 A*68:01	B*38:01 B*39:02	C*07:02 C*12:03	DRB1*07:01 DRB1*14:02	DQB1*02:02 DQB1*03:01	DPB1*04:02 DPB1*71:01	DQA1*02:01 DQA1*05:03	Not tested	**10**	*2*	**3**	*1*	**7**	*1*
A*01:01 A*68:02	B*08:01 B*53:01	C*06:02 C*07:01	DRB1*03:01 DRB1*14:54	DQB1*02:01 DQB1*05:03	DPB1*01:01 DPB1*01:01	DQA1*01:04/05 DQA1*05:01	Not tested	**6**	*1*	**4**	*1*	**5**	*2*
A*02:01 A*02:01	B*07:02 B*35:17	C*04:01 C*07:02	DRB1*14:06 DRB1*15:01	DQB1*03:01 DQB1*06:02	DPB1*04:01 DPB1*04:01	Not tested	DPA1*01:03 DPA1*01:03	**57**	*4*	**43**	*1*	**54**	*5*
A*02:01 A*02:06	B*39:02 B*39:05	C*03:04 C*07:02	DRB1*04:04 DRB1*08:02	DQB1*03:02 DQB1*04:02	DPB1*04:01 DPB1*04:02	Not tested	DPA1*01:03 DPA1*01:03	**57**	*3*	**36**	*3*	**49**	*4*
A*02:01 A*24:02	B*40:01 B*40:01	C*03:04 C*07:02	DRB1*04:05 DRB1*13:02	DQB1*04:01 DQB1*06:04	DPB1*02:01 DPB1*05:01	Not tested	DPA1*01:03 DPA1*02:02	**54**	*6*	**32**	*6*	**47**	*7*
A*02:01 A*29:02	B*35:01 B*44:03	C*04:01 C*16:01	DRB1*07:01 DRB1*14:02	DQB1*02:02 DQB1*03:01	DPB1*04:01 DPB1*06:01	DQA1*02:01 DQA1*05:04	Not tested	**66**	*6*	**40**	*2*	**47**	*5*
A*02:01 A*68:01	B*40:08 B*44:03	C*03:04 C*16:01	DRB1*04:07 DRB1*07:01	DQB1*02:02 DQB1*03:02	DPB1*04:02 DPB1*11:01	Not tested	DPA1*01:03 DPA1*02:01	**48**	*5*	**25**	*1*	**39**	*3*
A*02:03 A*32:01	B*38:02 B*40:01	C*07:02 C*07:02	DRB1*14:05 DRB1*16:02	DQB1*05:02 DQB1*05:03	DPB1*02:02 DPB1*05:01	DQA1*01:02 DQA1*01:04	DPA1*02:02 DPA1*02:02	**67**	*3*	**50**	*3*	**59**	*5*
A*02:05 A*24:02	B*08:01 B*50:01	C*06:02 C*07:01	DRB1*07:01 DRB1*15:01	DQB1*02:02 DQB1*06:02	DPB1*04:01	DQA1*01:02 DQA1*02:01	Not tested	**75**	*7*	**43**	*6*	**60**	*9*
A*02:06 A*32:01	B*15:01 B*51:01	C*03:03 C*08:01	DRB1*04:04 DRB1*12:01	DQB1*03:01 DQB1*03:02	DPB1*04:01 DPB1*14:01	Not tested	DPA1*01:03 DPA1*02:01	**36**	*5*	**15**	*1*	**29**	*3*
A*02:07 A*34:01	B*39:15 B*46:01	C*01:02 C*12:02	DRB1*11:01 DRB1*14:04	DQB1*03:01 DQB1*05:03	DPB1*02:01 DPB1*05:01	Not tested	DPA1*02:02 DPA1*02:02	**64**	*1*	**43**	*4*	**53**	*3*
A*02:33 A*33:01	B*14:02 B*51:01	C*03:04 C*08:02	DRB1*01:01 DRB1*16:02	DQB1*03:01 DQB1*05:01	DPB1*04:02 DPB1*09:01	Not tested	DPA1*01:03 DPA1*02:01	**47**	*7*	**20**	*2*	**38**	*4*
A*03:01 A*23:01	B*08:01 B*44:02	C*05:01 C*07:01	DRB1*03:01 DRB1*04:03	DQB1*02:01 DQB1*03:02	DPB1*01:01 DPB1*04:01	DQA1*03:01 DQA1*05:01	DPA1*01:03 DPA1*02:03	**9**	*2*	**7**	*4*	**10**	*1*
A*03:01 A*24:02	B*07:02 B*35:01	C*07:02 C*07:02	DRB1*14:02 DRB1*15:01	DQB1*03:04 DQB1*06:02	DPB1*04:01 DPB1*04:02	DQA1*01:02 DQA1*05:03	Not tested	**13**	*2*	**7**	*5*	**10**	*2*
A*11:01 A*11:01	B*15:35 B*15:35	C*07:02 C*07:02	DRB1*15:02 DRB1*15:02	DQB1*05:02 DQB1*05:02	DPB1*05:02 DPB1*05:02	DQA1*01:02 DQA1*01:02	DPA1*02:02 DPA1*02:02	**11**	*1*	**5**	*4*	**7**	*0*
A*11:01 A*29:02	B*51:01 B*51:01	C*03:03 C*16:01	DRB1*07:01 DRB1*13:01	DQB1*02:02 DQB1*06:03	DPB1*02:01 DPB1*11:01	DQA1*01:03 DQA1*02:01	DPA1*01:03 DPA1*02:01	**10**	*1*	**6**	*5*	**8**	*2*
A*23:01 A*30:04	B*40:01 B*51:01	C*03:04 C*15:02	DRB1*11:01 DRB1*16:01	DQB1*03:01 DQB1*05:02	DPB1*04:01 DPB1*04:02	Not tested	DPA1*01:03 DPA1*01:03	**10**	*3*	**6**	*1*	**9**	*1*
A*23:01 A*36:01	B*45:01 B*53:01	C*04:01 C*06:02	DRB1*11:01 DRB1*11:01	DQB1*06:02 DQB1*06:02	DPB1*02:01 DPB1*13:01	DQA1*01:02 DQA1*01:02	DPA1*01:03 DPA1*02:01	**11**	*1*	**5**	*2*	**9**	*2*
A*24:02 A*24:02	B*35:02 B*39:06	C*04:01 C*07:02	DRB1*03:01 DRB1*14:06	DQB1*02:01 DQB1*03:01	DPB1*02:01 DPB1*04:02	DQA1*05:01 DQA1*05:03	DPA1*01:03 DPA1*01:03	**11**	*3*	**6**	*4*	**8**	*2*
A*24:02 A*25:01	B*18:01 B*44:03	C*05:01 C*16:01	DRB1*03:01 DRB1*11:02	DQB1*02:01 DQB1*03:19	DPB1*04:01 DPB1*04:01	DQA1*05:01 DQA1*05:05	DPA1*01:03 DPA1*01:05	**8**	*0*	**5**	*3*	**7**	*1*
A*24:02 A*31:01	B*15:15 B*35:17	C*01:02 C*04:01	DRB1*04:10 DRB1*08:02	DQB1*03:02 DQB1*04:02	DPB1*03:02 DPB1*04:02	DQA1*03:01 DQA1*04:01	DPA1*01:03 DPA1*01:03	**7**	*2*	**4**	*3*	**5**	*2*
A*24:02 A*33:01	B*14:02 B*14:02	C*02:02 C*08:02	DRB1*01:02 DRB1*13:03	DQB1*03:01 DQB1*05:01	DPB1*03:01 DPB1*05:01	DQA1*01:01 DQA1*05:05	DPA1*01:03 DPA1*02:01	**11**	*1*	**6**	*4*	**8**	*3*
A*24:02 A31:01	B*35:01 B*40:02	C*03:04 C*04:01	DRB1*07:01 DRB1*14:02	DQB1*02:02 DQB1*03:01	DPB1*04:01 DPB1*11:01	Not tested	DPA1*01:03 DPA1*02:01	**9**	*3*	**5**	*1*	**8**	*2*
A*31:01 A*32:01	B*40:01 B*51:01	C*03:04 C*04:01	DRB1*03:01 DRB1*04:04	DQB1*02:01 DQB1*03:02	DPB1*04:01 DPB1*04:01	Not tested	DPA1*01:03 DPA1*01:03	**11**	*2*	**5**	*1*	**11**	*3*
A*33:01 A*68:01	B*14:02 B*48:01	C*08:01 C*08:02	DRB1*03:01 DRB1*08:02	DQB1*02:01 DQB1*04:02	DPB1*01:01 DPB1*04:02	DQA1*04:01 DQA1*05:01	Not tested	**12**	*2*	**4**	*0*	**10**	*3*
A*68:01 A*68:01	B*48:01 B*49:01	C*07:01 C*08:01	DRB1*08:02 DRB1*11:02	DQB1*03:19 DQB1*04:02	DPB1*03:19 DPB1*04:02	DQA1*04:01 DQA1*05:05	DPA1*01:03 DPA1*03:01	**13**	*2*	**7**	*6*	**10**	*2*
A*68:01 A*68:02	B*14:02 B*40:02	C*03:04 C*08:02	DRB1*04:04 DRB1*13:03	DQB1*03:01 DQB1*03:01	DPB1*02:01 DPB1*04:02	Not tested	DPA1*01:03 DPA1*01:03	**9**	*1*	**5**	*2*	**8**	*0*

PREVENTION OF GVHD BY THE TX200-TR101 TREG BATCHES USED IN THE SKIN TRANSPLANT MOUSE MODEL

The functional capacity of the TX200-TR101 batches used in the skin transplant experiment (Batch 1 and 2) was tested in a GvHD model. The evolution of body weights after injection of PBMCs with or without TX200-TR101 Treg Batches 1 or 2 is presented in Fig. S5.

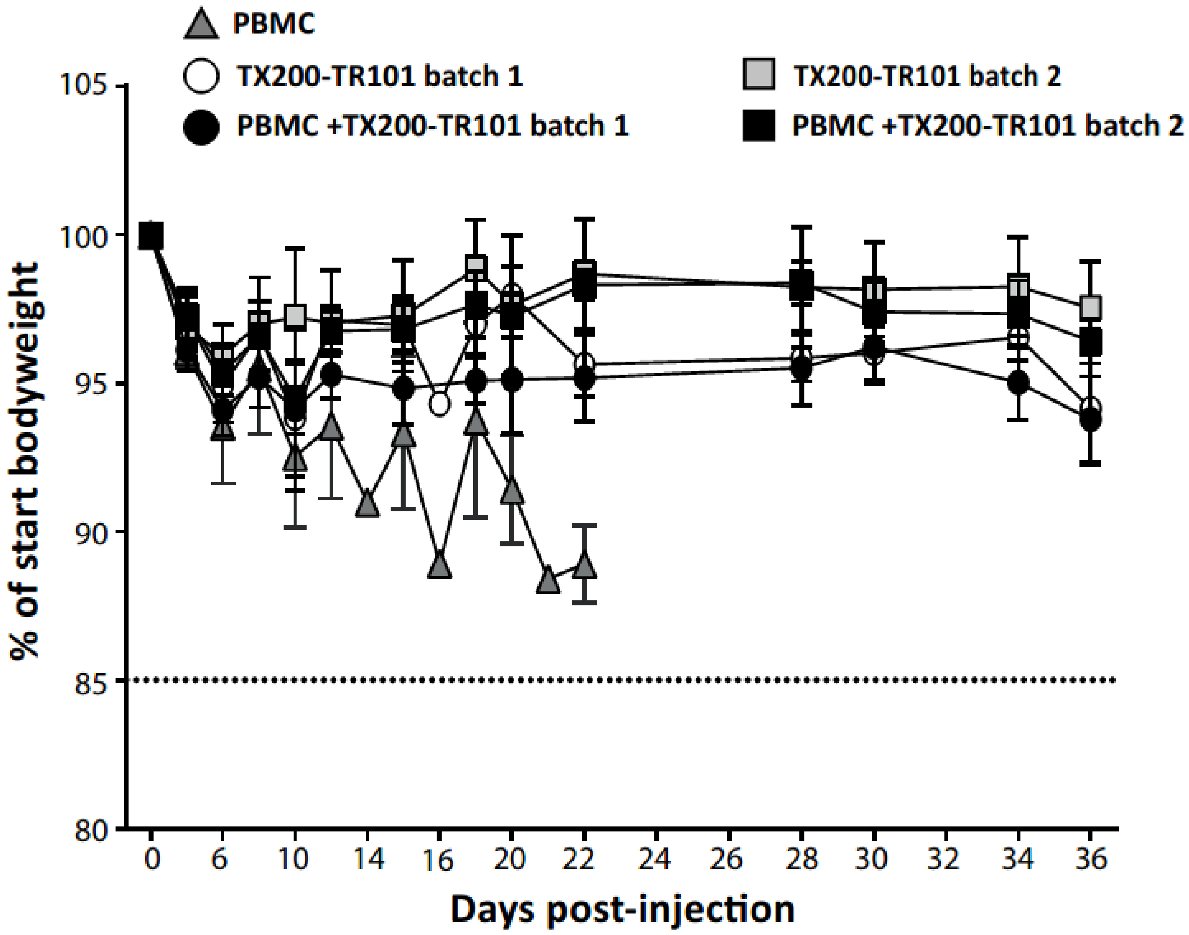

Fig. S5: Body weight changes post-delivery of PBMCs ± HLA-A*02 CAR Tregs (Batch 1 and 2) used in the skin transplant experiment.

Male and female 11 to 16-week old NSG mice were injected intravenously with HLA-A*02-positive human PBMCs with or without TX200-TR101 at a PBMC:Treg ratio of 2:1 (n=3 for PBMC; n=5 for TX200-TR101 alone, and n=4 for PBMC+TX200-TR101). Data are shown as mean ± SEM.

ANALYSIS OF NON-REGULATORY T CELL AND IMMUNE CELL CONTAMINANT

Detection and quantification of non-Tregs in 3 batches of TR101 Tregs (QR003, QR004, and QR005), un-transduced or transduced with a CAR (TX200), is shown in Table S4.

Table S4: Flow cytometric quantification of non-regulatory T cells in 3 batches of TX200-TR101

Test	Details/Cell Surface Marker	CAR	Results (%)		
			QR003	QR004	QR005
Hematopoietic stem cells	$CD34^+CD38^-$	CAR-	BLQ	BLQ	BLQ
		CAR+	BLQ	BLQ	BLQ
Myeloid progenitor cells	$CD34^+CD38^+CD123^+$	CAR-	0.012	0.017	ND
		CAR+	0.013	0.022	ND
Lymphoid progenitor cells	$CD34^+CD38^+CD127^+$	CAR-	0.001	0.000	ND
		CAR+	0.003	0.001	ND
Natural killer cells	$CD56^+$	CAR-	0.006	0.001	0.007
		CAR+	0.005	0.002	0.005
Lymphocyte B cells	$CD19^+$	CAR-	BLQ	BLQ	BLQ
		CAR+	BLQ	BLQ	BLQ
	$CD20^+$	CAR-	BLQ	BLQ	BLQ
		CAR+	BLQ	BLQ	BLQ
Monocytes	$CD14^+$	CAR-	0.001	0.000	0.001
		CAR+	0.000	0.000	0.001
Lymphocytes γδ	$CD3^+CD4^+$ $TCR\gamma\delta^+$	CAR-	0.011	0.005	ND
		CAR+	0.005	0.009	ND
Lymphocyte T cells	$CD8^+$	CAR-	BLQ	BLQ	BLQ
		CAR+	BLQ	BLQ	BLQ
Tconv	$CD3^+CD4^+CD127^{Hi}$	CAR-	BLQ	BLQ	BLQ
		CAR+	BLQ	BLQ	BLQ
iNKT cells	$CD3^+CD4^+$ $iTCR^+$	CAR-	0.001	0.000	ND
		CAR+	0.006	0.000	ND

BLQ: below limit of quantification; iNKT: invariant natural killer T cell; ND: not done; Tconv: conventional T cell
Results are expressed by percentage of this cell subset among Tregs measured by flow cytometry.
Note: BLQ indicates results below the limit of quantification of the MACSQuant Analyser 10 flow cytometer (Miltenyi Biotec);

Representative fluorescence-activated cell sorting (FACS) plots for 3 batches of TX200-TR101 (QR003, QR004, and QR005) for the analysis of $CD4^+$ and $FOXP3^+$ cells are shown in Fig. S6.

After gating on lymphocytes and gating out doublets (not shown), live human cells were identified using Live/Dead Viogreen dye. Expression of CD4 and FOXP3 was analysed to determine the percentage of $CD4^+FOXP3^+$ cells in each batch.

The gate for $CD4^+$ cells was set-up using the CD4 isotype control and the gate for FOXP3 was set-up using the FMO control (cells stained with all surface markers except FOXP3).

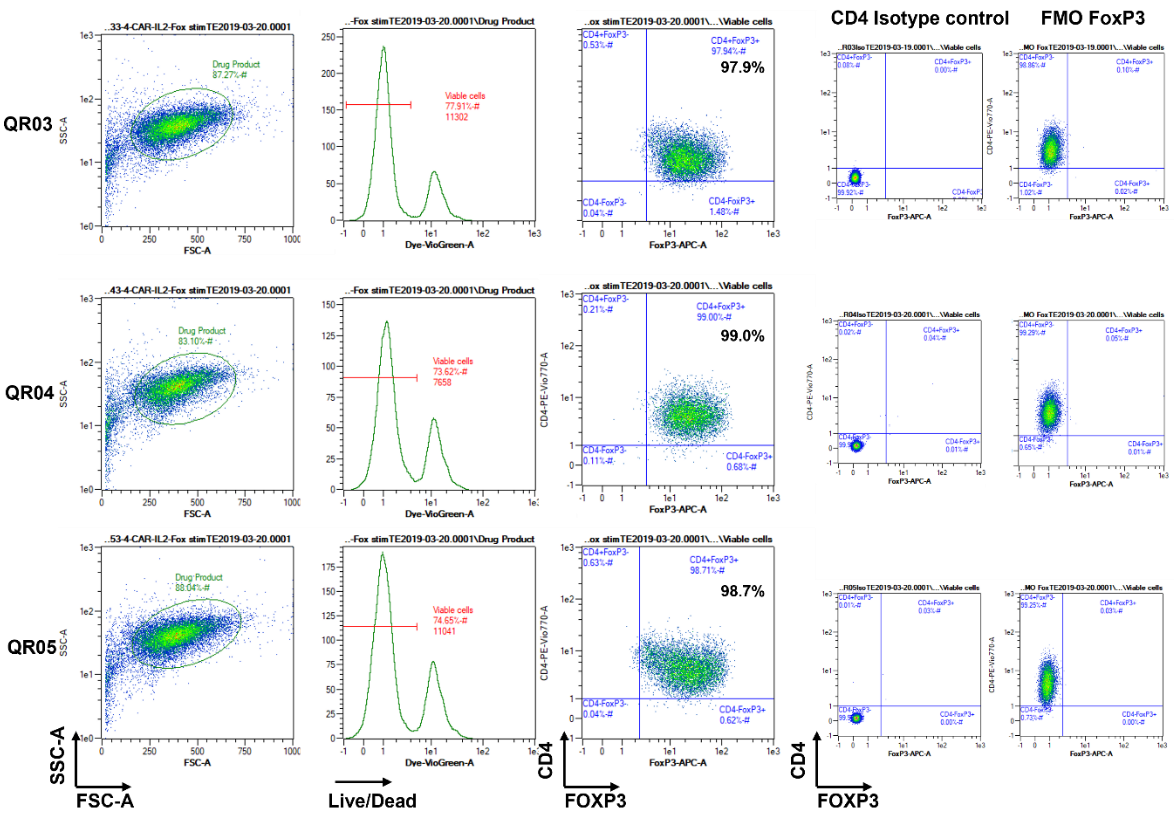

Fig. S6: Gating strategy for assessment of the purity of TX200-TR101 batches.

Representative cytofluorimetric plots showing the gating strategy to define human $CD4^+$ and $FOXP3^+$ cells

REFERENCES

1. Cooke K, Kobzik L, Martin T, Brewer J, Delmonte JJ, Crawford J et al. An experimental model of idiopathic pneumonia syndrome after bone marrow transplantation: I. The roles of minor H antigens and endotoxin. Blood. 1996;88:3230-39.
